# BioInstaller: a comprehensive R package to construct interactive and reproducible biological data analysis applications based on the R platform

**DOI:** 10.7717/peerj.5853

**Published:** 2018-10-31

**Authors:** Jianfeng Li, Bowen Cui, Yuting Dai, Ling Bai, Jinyan Huang

**Affiliations:** 1 State Key Laboratory of Medical Genomics, Shanghai Institute of Hematology, National Research Center for Translational Medicine, Ruijin Hospital, Shanghai Jiao Tong University School of Medicine, Shanghai Jiao Tong University, Shanghai, China; 2 School of Life Sciences and Biotechnology, Shanghai Jiao Tong University, Shanghai, China

**Keywords:** R package, Shiny application, Biological data analysis

## Abstract

The increase in bioinformatics resources such as tools/scripts and databases poses a great challenge for users seeking to construct interactive and reproducible biological data analysis applications. Here, we propose an open-source, comprehensive, flexible R package named BioInstaller that consists of the R functions, Shiny application, the HTTP representational state transfer application programming interfaces, and a docker image. BioInstaller can be used to collect, manage and share various types of bioinformatics resources and perform interactive and reproducible data analyses based on the extendible Shiny application with Tom’s Obvious, Minimal Language and SQLite format databases. The source code of BioInstaller is freely available at our lab website, http://bioinfo.rjh.com.cn/labs/jhuang/tools/bioinstaller, the popular package host GitHub, https://github.com/JhuangLab/BioInstaller, and the Comprehensive R Archive Network, https://CRAN.R-project.org/package=BioInstaller. In addition, a docker image can be downloaded from DockerHub (https://hub.docker.com/r/bioinstaller/bioinstaller).

## Introduction

With the rapid development of new bioscience technology, particularly next-generation sequencing (NGS), volumes of “omics” data have been generated, such as the 1000 Genomes Project, The Cancer Genome Atlas (TCGA), and Genotype-Tissue Expression (Abecasis et al., 2012; Cancer Genome Atlas Research Network et al., 2013; Lonsdale et al., 2013; Sanchez-Vega et al., 2018). The bioinformatics tools and databases required for the downstream data analysis are also increasing at a phenomenal rate. R language, as the most popular programming language for statistics, biological data analysis, and big data, has enabled diverse and free R packages (>14,000) for different types of applications, such as high-throughput sequencing data analysis (e.g., Bioconductor) (Gentleman et al., 2004) and the development of web applications (e.g., Shiny framework) (Chang et al., 2015). With the development of web technologies and the release of the R web developmental framework Shiny, the number of interfaces available to R users has increased. However, due to the lack of high-performance and open-source cloud platforms based on R (e.g., Galaxy for Python users) (Afgan et al., 2016), it is still difficult for R users, especially those without web development skills, to construct interactive and reproducible biological data analysis applications supporting the upload and management of files, long-time computation, task submission, tracking of output files, exception handling, logging, export of plots and tables, and extendible plugin systems.

Another common problem usually faced by R and other programming platform users (e.g., the team of Galaxy) (Afgan et al., 2016) is how to acquire and share certain bioinformatics resources quickly and accurately. Numerous bioinformatics tools (e.g., primer design, sequence alignment, variant calling and annotation) or scripts (e.g., data format conversion, text processing) are scattered around world web hosts. Biomedical databases are facing the same situation. For example, genome sequences (e.g., hg19/hg38 for human, mm9 and mm10 for mouse) are mainly deposited in the UCSC Genome Browser and National Center for Biotechnology Information (https://www.ncbi.nlm.nih.gov/) (Tyner et al., 2017). The best-known gene and transcript annotation resources are provided by GENCODE and the RefSeq database (Derrien et al., 2012; O’Leary et al., 2016). Genetic variants annotation databases, mainly cancer and Mendelian disorder related, are hosted by the original projects (for example, TCGA) and various down-stream tools (for example, ANNOVAR, Variant Effect Predictor and Oncotator) (McLaren et al., 2016; Ramos et al., 2015; Wang, Li & Hakonarson, 2010). Bioconductor is a popular bioinformatics R community for sharing genetic variants and other types of bioinformatics annotation databases via R package (Gentleman et al., 2004), but it is difficult for users to share many types of tools/scripts and databases if they do not have the capability of packing their own tools/scripts and databases. In most cases, these resources are isolated and can only be accessed via a command line tool such as rsync (https://rsync.samba.org/) or wget (http://www.gnu.org/software/wget/), to request the corresponding uniform resource locators (URLs). Software distribution tools that do not demand root privileges, such as conda (https://github.com/conda/conda) and spack (Gamblin et al., 2015), have greatly improved the acquisition of bioinformatics software. However, considering the huge growth of tools/scripts and databases required for bioinformatics data analysis, the resources supported by these software distribution tools are far from sufficient. Users also need more experience to use these different package management tools under command line environment.

Here, we present an open-source, comprehensive, flexible bioinformatics platform named BioInstaller that can be used to collect, manage and share various types of bioinformatics resources and to perform interactive and reproducible data analyses. By utilizing a simplified and standard Tom’s Obvious, Minimal Language (TOML) format configuration file with extra parse functions, the developers and users can freely and unreservedly share their public or internal bioinformatics tools/scripts and databases online on the GitHub repository or other hosts. In addition, users can easily obtain access to pooled bioinformatics resources via the diverse interfaces of BioInstaller, which includes R functions, the Shiny application (Chang et al., 2015) and HTTP representational state transfer (REST) application programming interfaces (APIs) that are rarely adopted in other similar tools. As a practical demonstration, we collected 157 tools/scripts and 110 databases specifically related to genetic variants annotation using the BioInstaller-defined configuration files. Notably, we developed a Shiny application to support functions including system monitoring, the logging system, file management, the queue system, and so on. This application can easily be reused in other Shiny applications. We expect the BioInstaller package and the practices in this work to reduce the difficulty of constructing the interactive and reproducible biological data analysis applications for R users, and to further improve the interactivity and reproducibility of bioinformatics data analysis.

## Materials and Methods

### Design and development of BioInstaller

**Figure 1:**
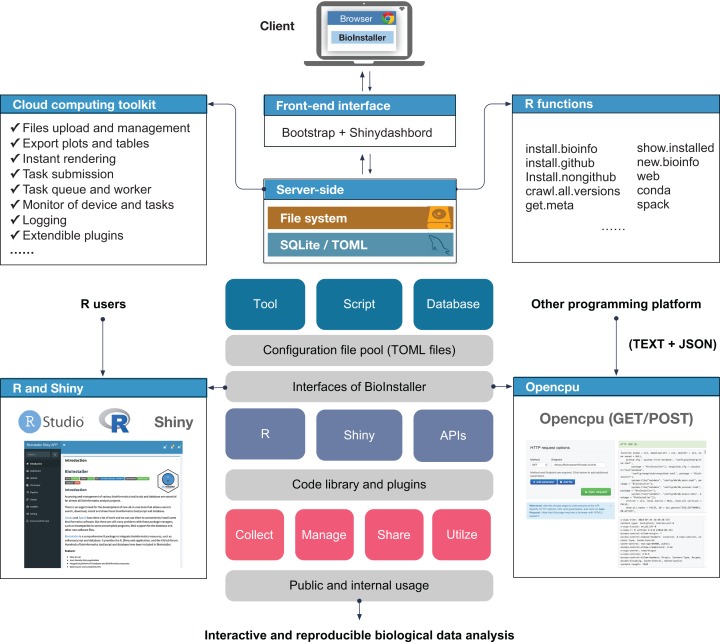
Overview of structure and functions of BioInstaller. Bioinformatics tools, scripts and databases are supported by BioInstaller. Bootstrap and Shinydashbord are used to construct the front-end interface. The R functions, Shiny and Opencpu services and the SQLite and TOML databases were applied in the back-end.

BioInstaller was designed as an interactive R package to collect, manage, and share various types of bioinformatics resources and perform interactive and reproducible data analyses. BioInstaller contains the R functions and the Shiny application (Chang et al., 2015) and REST APIs (Fig. 1). Both R and other programming platform users can utilize the functions of BioInstaller, such as by downloading bioinformatics tools/scripts and databases and performing statistical analysis and visualization. The R and Shiny interfaces of BioInstaller were mainly developed in R language and utilize the HTML/CSS and JavaScript languages. To run an instance of BioInstaller, the R program and extra dependent R packages are required. Travis CI (https://www.travis-ci.org/) was used to automatically test the R functions on Linux and MAC OSX platforms. Periodically, the tested and updated BioInstaller package is submitted to Comprehensive R Archive Network (CRAN) with an increased version number, for example, from v3.3.3 to v3.3.4. Both the open and restricted bioinformatics resources can be integrated using the TOML format configuration file. The configuration files can also be used in other programming language platforms to access desired masteries by using a unique item name, such as “bwa,” “gatk,” “annovar,” “db_annovar_1000g,” “db_annovar_gtex,” etc. A hash value was generated using the item name and version for the unique ids of tools/scripts and databases. An autogenerated docker image containing all required R packages and the backend web service of BioInstaller have been deposited at the DockerHub (https://hub.docker.com/r/bioinstaller/bioinstaller).

### GitHub API and custom values/functions for querying of version

The querying of versions of bioinformatics tools/scripts and databases of a GitHub or non-GitHub project is the basic function of BioInstaller. For GitHub projects items, the GitHub APIs were used to access the projects version information, such as release, tags, and branches. All released versions will be used as the available versions and returned to BioInstaller (Fig. S1A). However, the situation becomes more complicated if the resources have not been published on GitHub. Here, we propose two types of methods of parsing item versions. Method I: If the released versions are fixed, users can write it in the “version_available” field in the configuration file. Method II: Utilizing the R packages rvest (https://CRAN.R-project.org/package=rvest) and RCurl (https://CRAN.R-project.org/package=RCurl), we established an R functions pool to dynamically query the version of items from the original release website (Dataset S1). The demo function to query the latest version of GMAP is shown in Fig. S1B. This is useful for automating a pipeline to build the precompiled binary version.

### Mirror resource for an invalid link

Network transferring is a common problem in bioinformatics data analysis. A mirror resource is one option to partially resolve these problems, including an invalid link and network blocking. BioInstaller allows users to set any numbers of mirror URLs for their tools/scripts and databases to avoid the possible problems caused by network transmission. As shown in Fig. S1C, the mirror URLs of Miniconda (https://conda.io/miniconda.html) are separately provided by the official and our hosts. Notably, established mirror URLs of bioinformatics resources can be used in the spack (Gamblin et al., 2015) and other similar tools to build the cache files.

### TOML format configuration files

Massive bioinformatics tools/scripts and databases have been integrated into BioInstaller. TOML is a popular and human-readable configuration formats supporting comments. We uses standard TOML format configuration file to store required information of the included bioinformatics tools/scripts and databases. These configuration files can be reused in other bioinformatics software packages or data analysis pipelines via online accession or as a file copy. We have provided six directories to store different types of TOML files including “github,” “nongithub,” “database,” “web,” “docker,” and “shiny.” Due to the broad compatibility of BioInstaller, any resource published on docker, GitHub, Zenodo (https://zenodo.org/) or other platforms can be supported.

### Implementation of the Shiny application

To increase the convenience of BioInstaller for nonprogramming users, a user-friendly web application was developed based on Shiny (Chang et al., 2015). The user-interface (UI) of BioInstaller was constructed using the R package shinydashboard (https://cran.r-project.org/package=shinydashboard) and Shiny (Chang et al., 2015). Output tables were generated by the R package DT (https://CRAN.R-project.org/package=DT) and wrapped JavaScript library DataTables (https://datatables.net/). Charts were mainly generated by published R packages and in-house scripts or R packages that all support interactive update and export of PDF, SVG, and PNG format plots. The tab items of the BioInstaller Shiny application at the left side of the navigation bar can be used to switch among all available modules, including “Introduction,” “Dashboard,” “Upload,” “File Viewer,” “Pipeline,” “Instant,” “Installer,” and “Setting.” The detail usage guidelines are provided on our host (http://bioinfo.rjh.com.cn/labs/jhuang/tools/BioInstaller/), and R users can also use the browser vignettes functions in R to access these documents.

## Results

### Overview and practices of BioInstaller’s functionalities

**Figure 2:**
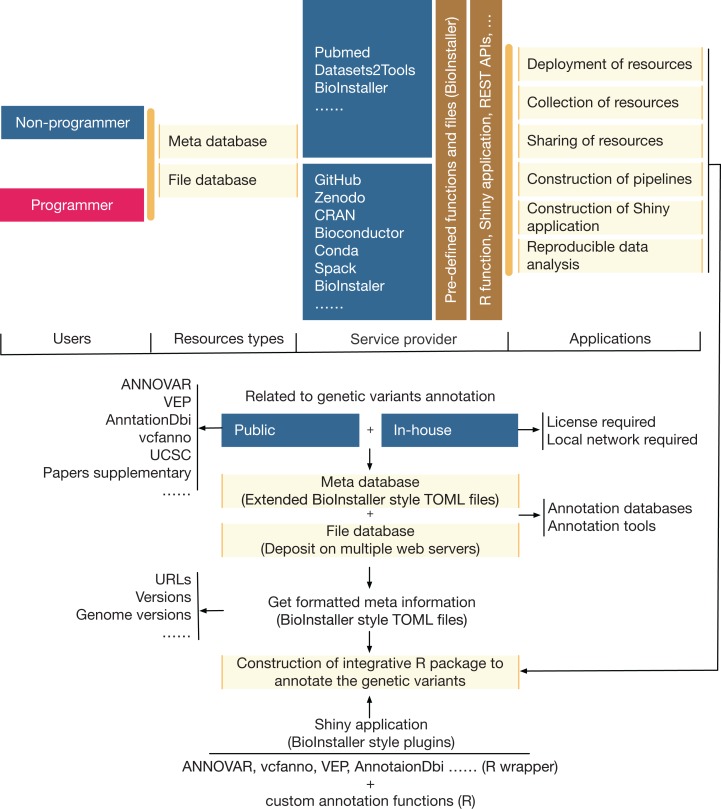
The relevance, applicability, and a real example of BioInstaller.

A comprehensive R package was developed that could be used to quickly construct interactive and reproducible biological data analysis applications based on the R platform (Fig. 2). The functionalities (Table 1; Dataset S2) of BioInstaller were divided into six parts based on whether users use BioInstaller or not: (1) deployment of resources, (2) collection of resources, (3) sharing of resources, (4) construction of pipelines, (5) construction of Shiny applications, and (6) reproducible data analysis. An example of a real project (annovarR, https://github.com/JhuangLab/annovarR, under development) is shown in Fig. 2 to illustrate the full workflow for BioInstaller utilization, which was designed to integrate various genetic variant annotation and visualization tools, including public command line tools, R packages and custom annotation and visualization functions. Using the code library, predefined TOML files (database resources and plugins), and the docker file of BioInstaller, we could easily customize the BioInstaller-established Shiny application to work on the genetic variants annotation tasks. If BioInstaller is not used, we need to develop the UI and server code of the Shiny application for a large number of universal functions, such as the file management system, background task submission and queue management, and tracking of the output log and files. The docker image of BioInstaller is also out-of-the-box and could be modified and applied to our own works. Based on the integrated installer (e.g., conda, spack, and BioInstaller) and simplified TOML files of BioInstaller, users can collect, share, and deploy genetic variant annotation databases and tools with one-stop service. As a real practice of BioInstaller, we collected and shared tools/scripts and databases in the configuration pool of BioInstaller, including genetic variant annotation databases and tools; the meta information is freely available and hosted on the public GitHub website (https://github.com/JhuangLab/BioInstaller/tree/master/inst/extdata/config). The raw files are stored on the original websites (e.g., https://github.com, https://sourceforge.net/, http://annovar.openbioinformatics.org/, etc.) and our host.

**Table 1:** List of the relevance and applicability of BioInstaller.

	With bioInstaller	Without bioInstaller
**Deployment of resources**		
User-interfaces	R functions, Shiny UI, REST APIs (Conda, Spack, and other tools/scripts)	Command-line tools (Conda, Spack, and custom tools)
Retrieve installed packages	Integrated Shiny dashboard page including R packages, conda and Python packages, Spack packages, and BioInstaller resource	Multiple command line operations
**Collection of resources**		
Local development	Yes	No
Need to register an account	Not need	Need
Type of backend databases	Default use TOML and SQLite (potable purpose) Plugins for other types	MySQL
**Resources hosts**	No limitation	Centralized
File sizes	No limitation	Limited
PubMed query	Integrated R codes with secret key (no limited access) Shiny UI with formatted table	Isolated R codes without secret key (limited access, *n* <= 20) Online version without formatted table
**Sharing of resources**		
Medium	Simplified TOML format files	Form or configuration file required more skills
Download service	Local Shiny application	Centralized web service or command line tools
**Construction of pipelines**		
Store of meta information (e.g., URL and version)	Pre-defined TOML file	*De novo* source code (e.g., ANNOVAR and fusioncatcher)
**Construction of Shiny application**		
Pre-defined pages	Pre-defined Shiny UI and server (Dashboard, file management, task submission, logging, export, and update of plots exception handling, setting)	Isolated examples UI and server codes
Difficulty	Easy to construct the Shiny application (Plugins + optional R codes)	Relatively complicated (Require R codes for UI and server)
**Reproducible data analysis**		
Logging	Support	Manual
Docker image	Pre-defined docker image with Shiny, Rstudio, and Opencpu services	Most not

### Comparison of BioInstaller with existing tools for the collection and sharing of bioinformatics resources

To better understand the advance provided by BioInstaller in terms of the collection and sharing of bioinformatics resources, we further compared BioInstaller with several existing tools, including Omictools (Henry et al., 2014) and Datasets2Tools (Torre et al., 2018) (Tables 1 and 2), the two most comprehensive meta databases focused on bioinformatics tools. All provide a web forum to update the meta database of bioinformatics resources. However, BioInstaller offers an off-line way to develop the users’ own meta databases via an unlimited configuration file pool (TOML and SQLite format) that is easy to carry and share and is independent of programming knowledge. In addition, the developed R functions and Shiny application can be used to query and download the linked or isolated file databases, such as appendix data from papers, annotation databases for genetic variants, genome sequences, etc. In most cases, it is suitable to tightly combine the meta database with the file database. Therefore, we designed and shared an upload module in the Shiny application to set the meta information for all files, and users can add the description, genome version, custom file types, and other customizable fields. Both Omictools and Dataset2Tools only include the items in their databases and do not integrate external resources. BioInstaller not only can be used to collect users own resources, but also can be used to integrate external resources.

**Table 2:** Overview comparison of BioInstaller and existing tools on the collection and share of bioinformatics resources.

	BioInstaller	Omictools	Datasets2Tools
**Infrastructure and utilities**			
Programing language	R, JavaScript	HTML/CSS/JavaScript	HTML/CSS/JavaScript
Chrome extension	No	No	Yes
Web service	R Shiny	Web	Web
R functions	Yes	No	No
REST APIs	Yes	No	Yes
Backend database	TOML and SQLite	Not available	MySQL
Docker image	Yes	No	No
**Functionality**			
Access and collect meta database	Yes	Yes	Yes
Access and collect file database	Yes	No	No
Integration of external resources	Yes	No	No
PubMed query	Yes	No	No
Dataset query	Yes	No	Yes
Number of supported resources	Integrated	High	Medium
Version query	Yes	No	No
Download service	Yes	No	No
Local branch and development	Yes	No	No
**Input and output**			
Input	R functions, Web text, APIs	Web text only	Web text + APIs
Output	Text, table, plots, and Web page (PNG, SVG and PDF)	Web page	Text and web page

### Summary of supported bioinformatics tools/scripts and databases

For now, 157 tools/scripts and 110 databases are natively supported in BioInstaller (Fig. 1 and Table 3; Tables S1 and S2). First, we covered the most commonly used tools in each bioinformatics analysis process, including data quality control (*n* = 17), alignment and assembly (*n* = 27), variant detection (*n* = 32) or annotation (*n* = 12), high-throughput sequence manipulation (*n* = 17), and visualization libraries (*n* = 11) (Table 3; Table S1), etc. Second, BioInstaller also provides abundant databases for annotating data or satisfying software dependencies. With BioInstaller, users can easily download UCSC sequence and annotation data (*n* = 4,995) (Dataset S3), blast databases (*n* = 29) (Table S2), allele frequency databases (*n* = 17), variant effect prediction databases (*n* = 29), and disease-related (*n* = 13), drug-related (*n* = 4), noncoding region-related databases (*n* = 15) (Table 3; Table S2), among others. Notably, we collected and constructed 20 genetic variant annotation databases, which can be directly used in other variants annotation tools, including ANNOVAR (Wang, Li & Hakonarson, 2010), vcfanno (Pedersen, Layer & Quinlan, 2016), and annovarR (https://github.com/JhuangLab/annovarR).

**Table 3:** Summary of BioInstaller included tools/scripts and databases.

Category	Number
**1, Tools/scripts**	
Alignment and assembly	27
Quality control	17
HTS manipulation	17
Association analysis	6
Genetic variants annotation	12
Detection of SNVs, INDELs and SVs	32
Immunity-associated	2
Isoform analysis	3
Gene expression analysis	9
Network analysis	3
Visualization libraries	11
System dependence	18
**2, Databases**	
Variant-level	
Allele frequency	17
Variants Effect prediction	29
Disease-related	6
Gene-level	
Basic information	8
Gene function	3
Disease-related	7
Drug related	4
Noncoding RNA related	15
Reference genome	9
Protein related	4
Others	8

BioInstaller has been released on CRAN for one and a half years and has accumulated a certain number of users, with a total of 19,912 downloads from CRAN (2018.8.3). In the recent release (v0.3.5), we provided the Shiny application and significantly expanded the supported tools/scripts and databases. The number of supported tools/scripts and databases is still increasing and is being applied to other related projects, such as the integrated genetic variants annotation tool annovarR (https://github.com/JhuangLab/annovarR).

### Examples of BioInstaller R functions

We have demonstrated the basic structure, functions, and web service of BioInstaller. The full help document is available at http://bioinfo.rjh.com.cn/labs/jhuang/tools/BioInstaller/articles/. Because most of the Shiny application UIs are wrapped with R functions, we use several use examples to illustrate the R functions of BioInstaller.

**Example #1:** Install packed or unpacked bioinformatics tools. We use the Ion Torrent Variant Caller (Zook et al., 2014) and svaba (Wala et al., 2018) to show how to install or download the bioinformatics tools or scripts that are not supported by other package management tools.

> library(BioInstaller) # Library the R package
> set.biosoftwares.db(“∼/.BioInstaller/info.yaml”) # Store the installation information
> install.bioinfo(show.all.names = TRUE) # Get all items name supported by BioInstaller
> install.bioinfo(name = “tvc,” show.all.versions = TRUE) # Get all available versions of tvc
> install.bioinfo(name = “svaba,” show.all.versions = TRUE) # Get all available versions of svaba
> install.bioinfo(name = “tvc,” download.dir = “/path/tool/tvc”) # One-click install the tvc
> install.bioinfo(name = “svaba,” download.dir = “/path/tool/svaba”) # One-click install the svaba
> show.installed() # Get all installed tools
> get.info(“svaba”) # Get the svaba installation information, such as update time and version

**Example #2:** Download genetic variants annotation databases. Genetic variants annotation is a common and high-demand task for most biomedicine research, especially for examining the correlations between phenotype and molecular features, such as germline and somatic mutations. The followed example describes how to download the genetic variants annotation databases dbSNP, CIViC, DisGeNET, and CancerHotspot (Chang et al., 2016; Griffith et al., 2017; Piñero et al., 2017).

> install.bioinfo(“db_annovar_avsnp,” extra.list = list(buildver = “hg19”), download.dir = “/path/db/”) # install the latest dbSNP from ANNOVAR website
> crawl.all.versions(“db_annovar_avsnp”) # Download all dbSNP to current directory
> install.bioinfo(“db_civic,” download.dir = “/tmp/db”) # Download the nightly version of CIViC database
> install.bioinfo(“db_disgenet,” download.dir = “/tmp/db”) # Download the DisGeNET database
> install.bioinfo(“db_cancer_hotspots,” download.dir = “/tmp/db”) # Download the DisGeNET databaseß

**Example #3:** Download an annotation database based on the supplementary files of published papers. The followed example is an epigenetic genes classification (e.g., reader, writer, eraser) database only available in the papers supplementary file (Huether et al., 2014).

> install.bioinfo(“db_annovar_epi_genes,” extra.list = list(buildver = “hg19”), download.dir = “/path/db/”) # install the epigenetic genes database from our website

### User-interfaces and functions of the Shiny application

#### Introduction module

Utilizing the Shiny function “includeMarkdown,” we generated the “Introduction” module page from Markdown, a lightweight markup language, format document (Fig. S2A).

#### Dashboard module

The “Dashboard” module includes the system monitors, such as hardware (Disk and memory), queue tasks, task log, installed R packages, Python packages, conda environments, and the other information of the operating environments (Figs. S2B, S2C and S3). The monitored data stream is automatically updated once every 10 s (Fig. S2B). A demo table output in the dashboard lists all files in the environment variable “PATH,” where users can use the selector at the lower left quarter to customize the row numbers (5, 10, 25, 50, and all) (Fig. S2C). All output tables in BioInstaller can be easily exported to CSV, XLS, PDF files or directly copied to the clipboard. Monitor plugins related to the information of the R system (Figs. S3A, S3B and S3C), conda (Fig. S3D), BioInstaller (Fig. S3E), and spack (Fig. S3F) are integrated in this work, which can reduce user input of extra command line commands and facilitate sharing with others.

#### Upload module

The “Upload” module is used to upload files to the BioInstaller Shiny web platform. Optional fields, such as file type, genome version, and description, can be stored in the SQLite format database with the uploaded files path and the files md5 value (Fig. S2D). When uploading a file, users need to click the “Save” button to confirm the upload behavior and update the database (Fig. S2E). Before the confirmation click, users can preview the file and make a final decision (Fig. S2F). Files with sizes ranging from 0.25 to 8 GB were tested on the Shiny application (Table S3). For files larger than 10 GB, we recommend using the rsync or FTP service to transfer files and then adding the corresponding description and records in TOML or SQLite databases.

#### File viewer module

**Figure 3:**
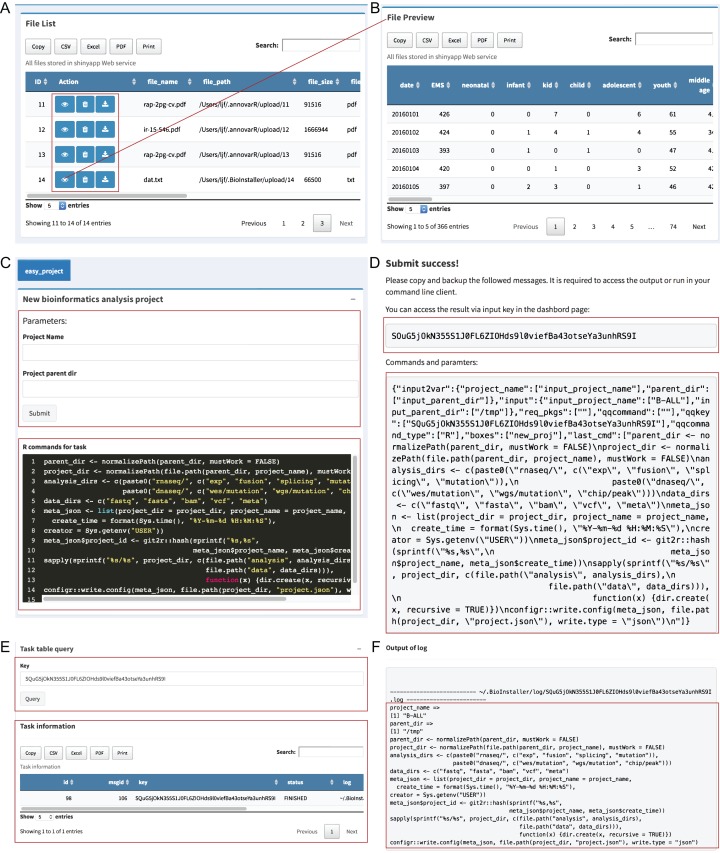
Shiny application modules of file viewer and pipeline. (A) Uploaded files are showed on the table where view, deletion, and download function are provided. (B) The interface of the preview result. (C) Easy project was used as the demo in pipeline module, which could be used to create a series of directories via submitting a queue task with two parameters: project name and parent directory. (D) The dialog box displays a prompt message with a queue character key. (E) Task queue and queue information can be requested by the character key in the dashboard module. (F) Function to get the output log of the submitted task.

The “File viewer” module is used to manage all uploaded files in the BioInstaller Shiny application that supports view, delete and download, and all files can be used in the other plugins of the BioInstaller Shiny application, mainly in the “Pipeline” and “Instant” modules (Figs. 3A and 3B).

#### Pipeline module

The “Pipeline” module is used to integrate complicated bioinformatics analysis workflows or other small scripts. An in-house interpreter R function was used to parse the plugin configuration files to generate the Shiny UI and server functions. A small script creating a data analysis directory structure was used as the demo for “Pipeline” (Dataset S4). Users can input the project name and the parent directory to create a series of predefined directories. The R commands used in this task are editable at the bottom of the box (Fig. 3C). After users click the “Submit” button, BioInstaller will generate a random character as the submitted task key. Users can use this key to retrieve the output information, such as files and logs, in the “Dashboard” module (Figs. 3C–3F). All submitted tasks enter the task queue supported by the SQLite database using the R package litseq (https://CRAN.R-project.org/package=liteq). Tasks in the queue are automatically checked by the activated workers (Figs. 3E and 3F).

#### Instant module

**Figure 4:**
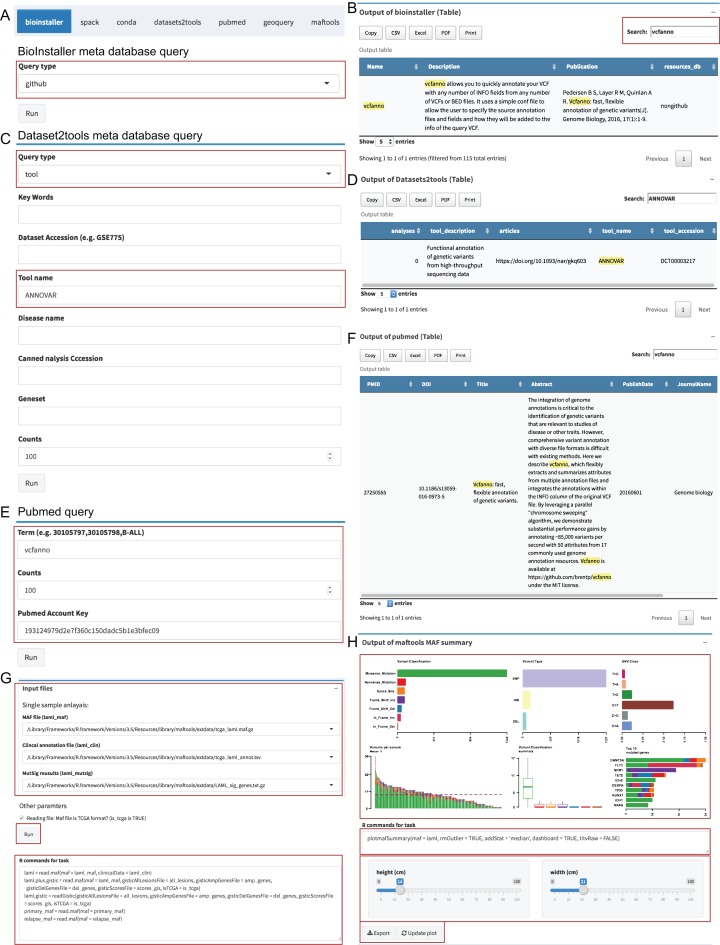
Shiny application “Instant” module. (A, B) The input box and output of BioInstaller Shiny plugin for querying the TOML format meta databases. (C, D) The input box and output of Dataset2tools Shiny plugin. (E, F) The input box and output of PubMed query Shiny plugin. (G) The input box of Maftools Shiny plugin. (H) One of Maftools demo output boxes which contains the plot, R command, export and update button.

The “Instant” module is used to run the real-time plots and data analysis, and similar to the “Pipeline” module, the UI and server were automatically generated via plugin configuration files (Dataset S5). We used the meta database query of BioInstaller, Datasets2tools (Torre et al., 2018), PubMed, and plots of Maftools (Mayakonda & Koeffler, 2016), a cancer somatic mutations visualization tool, as the demo to demonstrate the function. Users can select the input files defined in the plugins configuration file (TOML) or user-uploaded files. The commands are stored in the bottom of the boxes and can be modified by the user. After clicking the “Run” button, all output box codes, such as output plots and tables, run on the server side and are returned in real time to the Shiny UI (Fig. 4). We developed several plugins to query and access several meta databases related to bioinformatics, such as the BioInstaller meta databases (Figs. 4A and 4B), Datasets2tools (Torre et al., 2018) (Figs. 4C and 4D), and PubMed (Figs. 4E and 4F). The powerful visualization functions of R packages are also supported in the “Instant” module. As shown in Figs. 4G and 4H, users can obtain the demo output (PDF and PNG format) of Maftools. After running all box codes, a single box can be separately updated and exported by users.

#### Installer module

The “Installer” module is the main Shiny interface of BioInstaller for downloading and installing bioinformatics tools/scripts and databases. We provide the Shiny interfaces of BioInstaller, conda and spack (Fig. S4). The “Installer” module is similar to the “Pipeline” module, which is also needed to submit a task to the queue. The status and log information can be retrieved in the “Dashboard” module. Three basic use cases of the BioInstaller Shiny application are available: (1) download db_annovar_refgene database (Figs. S4A–S4B); (2) create conda environment (Figs. S4C–S4D); (3) install “zlib” using spack (Figs. S4E–S4F).

#### Setting module

The setting module is the interface for setting the value of the variable used in the BioInstaller plugins or R files. Both a Shiny UI and a YAML editor are offered for users (Figs. S5A and S5B). Any updates of the YAML editor (Fig. S5B) can change and refresh the Shiny UI options (Fig. S5A). It is helpful for users to manage various material related to BioInstaller and its plugins.

In most cases, through the one-click interface of BioInstaller, users can easily download and install the desired bioinformatics resources without any command line skills. Functions for automatic compiling from the source file with the dependent software or database are also supported in BioInstaller. However, for complicated software with high system dependence, we recommend using the interfaces of conda (https://conda.io/docs/) and spack (Gamblin et al., 2015).

### Portable message queue for background tasks based on SQLite

Tasks with long-time costs are challenging in Shiny, which always blocks the other interactive operations simultaneously when the previous task has not been finished. Here, we utilized the R package litseq (https://CRAN.R-project.org/package=liteq) to submit and manage the background queue tasks. litseq is portable and lightweight. litseq does not require extra software or service from other programming platforms and can work on any clusters server running computing-intensive tasks. The developed queue worker in BioInstaller can be used for all other background tasks submitted by litseq. All litseq-submitted tasks of BioInstaller are assigned a unique identification id. All executed commands, output logs, and others are saved in the permanent files.

### Opencpu backend service improves reproducibility

**Figure 5:**
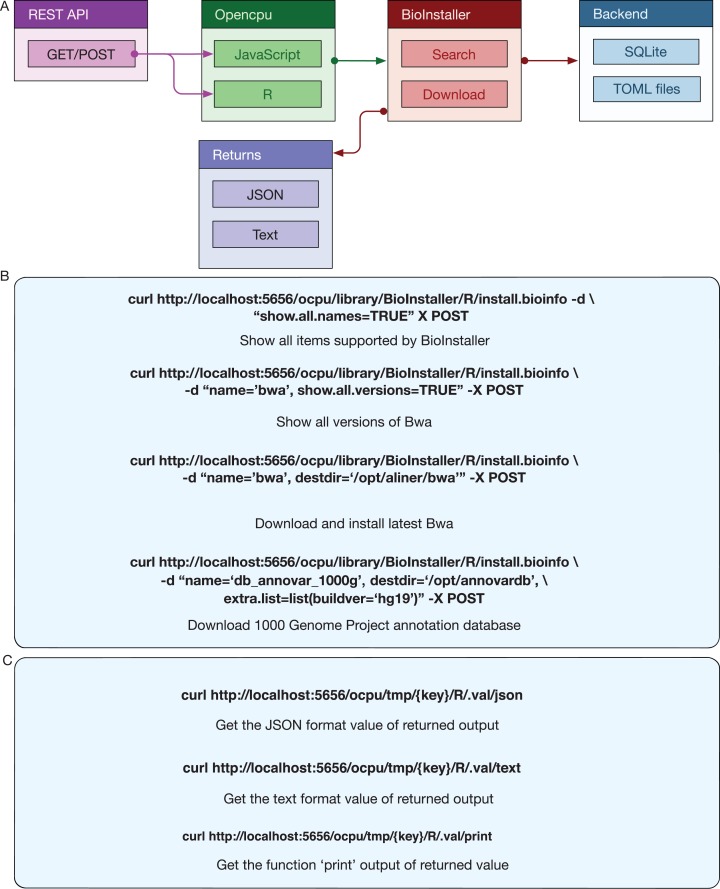
REST APIs of BioInstaller. (A) Workflow of REST APIs of BioInstaller that JSON and TEXT returns through the GET/POST query. (B) Using curl to invoke background R functions of BioInstaller. (C) The key character with GET method is provided to get the background R session output.

Opencpu (Ooms, 2017) is an R package for reproducible research that can expose a web REST API interface with R, Latex, and Pandoc. The R functions of BioInstaller are invoked by the activated Opencpu R process or daemon service. For other programming platform users, this is one possible method for utilizing the R functions of BioInstaller (Fig. 1). The output of JSON and text formats are returned when using the browser access (Fig. 5A) or simulated requests. Three of the most basic APIs usages of BioInstaller were used to demonstrate how it works: (1) obtaining all supported tools/scripts and databases; (2) acquiring available versions of the appointed item; (3) installing a tool in a directory (Fig. 5B). Notably, a random string, such as “x0a469794fa,” will be generated as the key of Opencpu to obtain the output of one R session. Both JSON and text format output can be returned by Opencpu backend APIs (Fig. 5C).

### Docker container of BioInstaller

A prebuilt docker image is available on the DockerHub (https://hub.docker.com/r/bioinstaller/bioinstaller), and the latest code change of the BioInstaller repository can automatically trigger an update of the docker image. In the docker image, we integrated and configured three types of web services, including Opencpu, Shiny (Chang et al., 2015; Ooms, 2017), and the RStudio server (https://www.rstudio.com/products/rstudio-server/). The followed commands can be used to deploy and start the service of BioInstaller service.

$ docker pull bioinstaller/bioinstaller
$ docker run -it -p 80:80 -p 8004:8004 bioinstaller/bioinstaller

Users can deploy a new instance host of BioInstaller and all other web services in a few minutes, and other tools/scripts and databases are also allowed to be embedded in this docker image using the Dockerfile (https://github.com/JhuangLab/BioInstaller/blob/master/Dockerfile).

### Use the GitHub forum to share, rate, and discuss the bioinformatics resources

The full-text search is natively supported by the GitHub website with highlight and age forwarding functions (Figs. S6A and S6B). To simplify the submitting of new items to BioInstaller, the GitHub repository issues page (https://github.com/JhuangLab/BioInstaller/issues) is recommended for other users to share, rate, and discuss bioinformatics tools/scripts and databases with a designated label (Fig. S6C). The “watching” function of GitHub can allow users to receive notifications of all conversations on the BioInstaller. Another advantage of establishing a free sharing community based on the GitHub is that all history changes on the code and forum posts can be recorded and retrieved. A rating function for bioinformatics tools/scripts or databases is also feasible by calculating the points corresponding to thumbs up or down.

## Discussion

Bioinformatics tools/scripts and databases are widely used in various data analysis projects. The construction of interactive and reproducible biological data analysis applications is critical for most bioinformatics data analyses (Henry et al., 2014; McQuilton et al., 2016; Ohno-Machado et al., 2017). The integrative utilization of these resources is becoming increasingly important for improving integrated biosciences data analysis. R language, as the most popular programming language for statistics, biological data analysis, and big data, has provided massive useful R packages for various data analysis efforts, especially the NGS field. However, there has been no comprehensive and free R application that can support file upload and management, perform long-time computation with a tasks submission system, track and record the output of files and log, develop extendible plugins, add or remove functions of the application in real time, and respond for REST APIs. Another common problem for users of R and other programming platforms for biological data analysis is that massive bioinformatics resources are isolated and scattered, which significantly increases the difficulty of deploying, collecting and sharing these resources. Well-known software distribution tools that do not need root privileges, such as conda (https://conda.io/docs) and spack (Gamblin et al., 2015), were designed for comprehensive fields and usually lack support for life science resources. Bioconda is a fine example of the centralized installation of bioinformatics software (approximately 1,900 items) that has significantly improved the reproducibility of bioinformatics data analysis (Gruning et al., 2018). However, this is not sufficient compared with the rapid increase in software and databases in the life sciences field.

As described in this study, we present a comprehensive, free and open-source platform, BioInstaller, to construct the interactive and reproducible biological data analysis applications. BioInstaller contains the R functions, the Shiny application, REST APIs and the docker image. This platform and the practices described in this work are sufficient for most R users to conveniently and quickly develop an interactive and reproducible biological data analysis application with diverse predefined functions (e.g., file management, task submission, plugin management system, logging, etc.), plugins, and files offered by BioInstaller. Moreover, based on the TOML format files, we have also integrated hundreds of bioinformatics resources required for the wide field of bioinformatics, such as sequence alignment, variant calling and annotation, and so on. We hope this newly presented open-source platform for R users can reduce the difficulty of constructing the interactive and reproducible biological data analysis applications and further improve the interactivity and reproducibility of bioinformatics data analysis.

## Conclusion

As described in this work, we established a new platform to construct interactive and reproducible biological data analysis applications based on R language. This platform contains diverse UIs, including the R functions and R Shiny application, REST APIs, and support for collecting, managing, sharing, and utilizing massive bioinformatics tools/scripts and databases.

## Supplemental Information

10.7717/peerj.5853/supp-1 Supplemental Information 1 List of BioInstaller included tools/scripts

10.7717/peerj.5853/supp-2 Supplemental Information 2 List of BioInstaller supported databases

10.7717/peerj.5853/supp-3 Supplemental Information 3 Time costs of upload and download files in Shiny application A set of files with file size range from 0.25 GB to 8 GB were used to test the performance of upload and download files in Shiny application.

10.7717/peerj.5853/supp-4 Supplemental Information 4 Demo functions and configuration item to query versions in BioInstaller (A) GitHub APIs integrated in R function can access GitHub projects meta information, including branches, tags and releases versions. (B) The demo function shows how to get the versions of the sequence alignment tool GMAP. (C) The demo configuration item shows how to download and install miniconda2 using mirror URL.

10.7717/peerj.5853/supp-5 Supplemental Information 5 Shiny application basic modules: introduction, dashboard and uploading (A) Overview of Shiny navigation bar tab items and the introduction module page. (B) Dashboard module page contains system monitor, task queue monitor, query system, and others. (C) Output table in dashboard module shows all files in ‘PATH’ directory’s environment variable. It also includes search and export functions. (D) UI of upload module contains extra fields, including file type, genome version, and description, to describe the information of the files. (E) Save button was used to save uploaded file and update the related database. (F) Preview box was used to view uploaded files before confirming uploading.

10.7717/peerj.5853/supp-6 Supplemental Information 6 More examples of dashboard module boxes (A) The example of R session information of host running Shiny application. (B) Installed R package of the host running Shiny application. (C) Output table of all environment variables and its value. (D)(E)(F)Output table and text of installed items via BioInstaller, conda, and spack.

10.7717/peerj.5853/supp-7 Supplemental Information 7 Shiny application ‘Installer’ module (A)(B) Using the download of ‘db_annovar_refgene’ shows how it works of Shiny ‘Installer’ module. Dynamic and interactive manipulations are supported. Log information of submitted download/install job can be recalled using given random characters. (C)(D) The input box and output log to create a new softwares environment using the conda plugin of ‘Installer’ module. (E)(F) The input box and output log to install ‘zlib’ using the spack plugin of ‘Installer’ module.

10.7717/peerj.5853/supp-8 Supplemental Information 8 Shiny application ‘Setting’ module (A) Rendered UI from the YAML format configuration file. (B) YAML editor allows users to add and modify variables used in the Shiny application. This can real-time update the UI of panel (A).

10.7717/peerj.5853/supp-9 Supplemental Information 9 Github forum of BioInstaller (A) Search the keyword on the github website with the the autosuggestor autocompletion functionality (B) Demo output of github search using ‘RNA’. Key word was colored yellow. (C) Demo posts of forum of BioInstaller.

10.7717/peerj.5853/supp-10 Supplemental Information 10 R functions pool of version query from original website (non-github)

10.7717/peerj.5853/supp-11 Supplemental Information 11 Management the environment of tools/scripts and databases, and the plugin system of BioInstaller

10.7717/peerj.5853/supp-12 Supplemental Information 12 Summary of UCSC sequence and annotation data which BioInstaller supported

10.7717/peerj.5853/supp-13 Supplemental Information 13 Demo plugin (easy_project) of ‘Pipeline’ module

10.7717/peerj.5853/supp-14 Supplemental Information 14 Demo plugin (maftools) of Shiny application ‘Instant’ module

## Additional Information and Declarations

### Data Availability

The following information was supplied regarding data availability: Project name: BioInstaller Project home page - Package: http://bioinfo.rjh.com.cn/labs/jhuang/tools/bioinstaller Project home page - Package (CRAN): https://CRAN.R-project.org/package=BioInstaller Project source code - Package: https://github.com/JhuangLab/BioInstaller. Project docker image: https://hub.docker.com/r/bioinstaller. Operating system(s): Platform independent Tested Operating system(s): MAC OS 10.13.6, Centos 7.1, Ubuntu 16.04, and Windows 10 (limited functions) Tested browsers: Google Chrome (v68), Safari (v11.1.2), and Firefox (v61.0.2) Programming languages: R, HTML, CSS and JavaScript Requirements: R (> 3.3.0), Shiny (R package), Opencpu (R package), and a web browser Optional functions: Maftools (R package), conda (Python package), spack (Python package), and docker License: MIT.
