# Supplementary material for: BioInstaller: a comprehensive R package to construct interactive and reproducible biological data analysis applications based on the R platform
Source: PeerJ. 2018 Oct 31;6:e5853. doi: 10.7717/peerj.5853 (PMC6215441; doi:10.7717/peerj.5853)
Supplement: Supplemental Information 1 [file peerj-06-5853-s001.docx]

| Tool Name | Description^a^ | URL | Category |
| --- | --- | --- | --- |
| Alignment and assembly | |  |  |
| ABySS | Assemble large genomes using short reads. | https://github.com/bcgsc/abyss | Genome, Transcriptome |
| bowtie | An ultrafast memory-efficient short read aligner. | https://github.com/BenLangmead/bowtie | Genome, Transcriptome |
| bowtie2 | A fast and sensitive gapped read aligner. | https://github.com/BenLangmead/bowtie2 | Genome, Transcriptome |
| BWA | Burrow-Wheeler Aligner for short-read alignment (see minimap2 for long-read alignment). | https://github.com/lh3/bwa | Genome |
| DART | A fast and accurate RNA-seq mapper with a divide-and-conquer strategy. | https://github.com/hsinnan75/DART | Transcriptome |
| hisat2 | A fast and sensitive alignment program for mapping next-generation sequencing reads (whole-genome, transcriptome, and exome sequencing data) to a population of human genomes (as well as to a single reference genome). | https://github.com/infphilo/hisat2 | Genome, Transcriptome |
| MARVEL | MARVEL consists of a set of tools that facilitate the overlapping, patching, correction and assembly of noisy (not so noisy ones as well) long reads. | https://github.com/schloi/MARVEL | Genome, Transcriptome |
| oases | De novo transcriptome assembler for short reads. | https://github.com/dzerbino/oases | Transcriptome |
| rHAT | A seed-and-extension-based noisy long read alignment tool. | https://github.com/HIT-Bioinformatics/rHAT | Genome, Transcriptome |
| RUM | An alignment, junction calling, and feature quantification pipeline specifically designed for Illumina RNA-Seq data. | https://github.com/itmat/rum | Transcriptome |
| SUBREAD | The Subread software package is a tool kit for processing next- generation sequencing data. It includes Subread aligner, Subjunc exon-exon junction detector and feature Counts read summarization program. Subread aligner can be used to align both gDNA-seq and RNA-seq reads. Subjunc aligner was specified designed for the detection of exon-exon junction. For the mapping of RNA-seq reads, Subread performs local alignments and Subjunc performs global alignments. | http://subread.sourceforge.net/ | Genome, Transcriptome |
| STAR | RNA-seq aligner. | https://github.com/alexdobin/STAR | Transcriptome |
| taxmaps | Large DNA/RNA metagenomics samples. | https://github.com/nygenome/taxmaps | Genome, Transcriptome |
| TMAP | Torrent mapping alignment program. | https://github.com/iontorrent/TS/tree/master/Analysis/TMAP | Genome, Transcriptome |
| tophat2 | A fast splice junction mapper for RNA-Seq reads. | https://github.com/infphilo/tophat | Transcriptome |
| GMAP | A Genomic Mapping and Alignment Program for mRNA and EST Sequences. | http://research-pub.gene.com/gmap/ | Transcriptome |
| MapSplice2 | Mapping RNA-seq data to reference genome for splice junction discovery that depends only on reference genome. | http://www.netlab.uky.edu/p/bioinfo/MapSplice2 | Transcriptome |
| NovoAlign | Powerful tool designed for mapping of short reads onto a reference genome from Illumina, Ion Torrent, and 454 NGS platforms. | http://www.novocraft.com/products/novoalign/ | Genome, Transcriptome |
| SSAHA2 | Sequence Search and Alignment by Hashing Algorithm. | http://www.sanger.ac.uk/science/tools/ssaha2-0 | Genome, Transcriptome |
| Velvet | Sequence assembler for very short reads. | http://www.ebi.ac.uk/~zerbino/velvet/ | Genome, Transcriptome |
| rMATS | MATS is a computational tool to detect differential alternative splicing events from RNA-Seq data. | http://rnaseq-mats.sourceforge.net/rmats3.2.5/ | Transcriptome |
| Olego | A crucial step in analyzing mRNA-Seq data is to accurately and efficiently map hundreds of millions of reads to the reference genome and exon junctions. | https://github.com/chaolinzhanglab/olego | Transcriptome |
| PRADA | Massively parallel sequencing of cDNA reverse transcribed from RNA (RNASeq) provides an accurate estimate of the quantity and composition of mRNAs. | https://sourceforge.net/projects/prada/ | Transcriptome |
| TrinityRNASeq | Trinity RNA-Seq de novo transcriptome assembly. | https://github.com/trinityrnaseq/trinityrnaseq | Transcriptome |
| EDENA | De novo short reads assembler. | http://www.genomic.ch/edena.php | Genome, Transcriptome |
| BLAST | Basic Local Alignment Search Tool. | https://blast.ncbi.nlm.nih.gov/Blast.cgi | Genome, Transcriptome |
| BLAT | A pairwise sequence alignment algorithm. | https://users.soe.ucsc.edu/~kent/src/ | Genome, Transcriptome |
| Quality control |  |  |  |
| ChronQC | A Quality Control Monitoring System for Clinical Next Generation Sequencing. | https://github.com/nilesh-tawari/ChronQC | Genome, Transcriptome |
| fastp | An ultra-fast all-in-one FASTQ preprocessor. | https://github.com/OpenGene/fastp | Genome, Transcriptome |
| fastx_toolkit | FASTA/FASTQ pre-processing programs. | https://github.com/agordon/fastx_toolkit | Genome, Transcriptome |
| MultiQC | A tool to create a single report with interactive plots for multiple bioinformatics analyses across many samples. | https://github.com/ewels/MultiQC | Genome, Transcriptome |
| FastQC | A quality control tool for high throughput sequence data. | http://www.bioinformatics.babraham.ac.uk/projects/fastqc/ | Genome, Transcriptome |
| PRINSEQ | A bioinformatics tool to pre-process and show Information of Sequence data. | https://sourceforge.net/projects/prinseq | Genome, Transcriptome |
| SolexaQA | A software to calculate quality statistics and visual representations of data quality for second-generation sequencing data. | https://sourceforge.net/projects/solexaqa | Genome, Transcriptome |
| seurat | R toolkit for single cell genomics. | https://github.com/satijalab/seurat | Single Cell |
| fastq-tools | Small utilities for working with fastq sequence files. | https://github.com/dcjones/fastq-tools | Genome, Transcriptome |
| NGS QC Toolkit | A Toolkit for Quality Control of Next Generation Sequencing Data. | http://www.nipgr.res.in/ngsqctoolkit.html | Genome, Transcriptome |
| PRADA | Massively parallel sequencing of cDNA reverse transcribed from RNA (RNASeq) provides an accurate estimate of the quantity and composition of mRNAs. | https://sourceforge.net/projects/prada/ | Transcriptome |
| TrimGalore | Trim Galore! is a wrapper script to automate quality and adapter trimming as well as quality control, with some added functionality to remove biased methylation positions for RRBS sequence files (for directional, non-directional (or paired-end) sequencing). | https://github.com/FelixKrueger/TrimGalore | Genome, Transcriptome |
| ContEst | A tool for estimating the level of cross-individual contamination in next-generation sequencing data. We demonstrate the accuracy of ContEst across a range of contamination levels, sources and read depths using sequencing data mixed in silico at known concentrations. | http://www.broadinstitute.org/cancer/cga/contest | Genome, Transcriptome |
| ABSOLUTE | The purpose of ABSOLUTE is to re-extract these data from the mixed DNA population. | http://archive.broadinstitute.org/cancer/cga/absolute | Genome |
| picard | A set of command line tools (in Java) for manipulating high-throughput sequencing (HTS) data and formats. | https://github.com/broadinstitute/picard | Genome, Transcriptome |
| HAPSEG | The HAPSEG module takes single nucleotide polymorphism (SNP) microarray data and outputs copy number data segmented by haplotype. The output data is suitable for use as input data for the ABSOLUTE module. | http://software.broadinstitute.org/cancer/software/genepattern/modules/docs/HAPSEG/1 | Genome |
| sequenza | A novel set of tools providing a fast python script to genotype cancer samples, and an R package to estimate cancer cellularity, ploidy, and genome wide copy number profile and infer for mutated alleles. | http://www.cbs.dtu.dk/biotools/sequenza/ | Genome |
| HTS manipulation | |  |  |
| bamtools | C++ API & command-line toolkit for working with BAM data. | https://github.com/pezmaster31/bamtools | Genome, Transcriptome |
| bamUtil | Some programs for working on SAM/BAM files. | https://github.com/statgen/bamUtil | Genome, Transcriptome |
| bedtools | The swiss army knife for genome arithmetic. | https://github.com/arq5x/bedtools2 | Genome, Transcriptome |
| bcftools | It contains all the vcf* commands which previously lived in the htslib repository (such as vcfcheck, vcfmerge, vcfisec, etc.) and the samtools BCF calling from bcftools subdirectory of samtools. | https://github.com/samtools/bcftools | Genome, Transcriptome |
| jvarkit | Java utilities for Bioinformatics. | https://github.com/lindenb/jvarkit | Genome, Transcriptome |
| seqtk | Toolkit for processing sequences in FASTA/Q formats. | https://github.com/lh3/seqtk | Genome, Transcriptome |
| vcflib | A simple C++ library for parsing and manipulating VCF files. | https://github.com/vcflib/vcflib | Genome, Transcriptome |
| vcftools | A set of tools written in Perl and C++ for working with VCF files. | https://github.com/vcftools/vcftools | Genome, Transcriptome |
| picard | A set of command line tools (in Java) for manipulating high-throughput sequencing (HTS) data and formats. | https://github.com/broadinstitute/picard | Genome, Transcriptome |
| htslib | HTSlib is an implementation of a unified C library for accessing common file formats, such as SAM, CRAM and VCF, used for high-throughput sequencing data, and is the core library used by samtools and bcftools. | https://github.com/samtools/htslib | Genome, Transcriptome |
| samstat | SAMStat displays various properties of next-generation sequencing reads stored in SAM/BAM format. | https://sourceforge.net/projects/samstat | Genome, Transcriptome |
| samtools | Tools (written in C using htslib) for manipulating next-generation sequencing data. | https://github.com/samtools/samtools | Genome, Transcriptome |
| sratools | The SRA Toolkit and SDK from NCBI is a collection of tools and libraries for using data in the INSDC Sequence Read Archives. | http://ncbi.github.io/sra-tools/ | Genome, Transcriptome |
| ucsc utils | Genome Browser and Blat application binaries built for standalone command-line use on various supported Linux and UNIX platforms. | http://hgdownload.cse.ucsc.edu/admin/exe | Genome, Transcriptome |
| BCL2FASTQ | Combines per-cycle BCL files from a run and translates them into demultiplexed FASTQ files. | https://support.illumina.com/downloads/bcl2fastq_conversion_software_184.html | Genome, Transcriptome |
| faToTwoBit | Convert DNA from fasta to .2bit format. | http://genome.ucsc.edu/goldenPath/help/blatSpec.html#faToTwoBitUsage | Genome, Transcriptome |
| liftOver | Converts genome coordinates and genome annotation files between assemblies. | https://genome.ucsc.edu/util.html | Genome, Transcriptome |
| Association analysis | |  |  |
| Beagle | Beagle is a software package for phasing genotypes and for imputing ungenotyped markers. | http://faculty.washington.edu/browning/beagle/beagle.html | Genome |
| BackSPIN | BackSPIN biclustering algorithm. | https://github.com/linnarsson-lab/BackSPIN | Transcriptome,  Single Cell |
| f-scLVM | Scalable and versatile factor analysis for single-cell RNA-seq. | https://github.com/PMBio/f-scLVM | Single Cell |
| RCA | An R package for robust clustering analysis of single cell RNA sequencing data. | https://github.com/GIS-SP-Group/RCA | Single Cell |
| BEARscc | Bayesian ERCC Assessment of Robustness. | https://github.com/Miachol/bearscc | Single Cell |
| sparsehash | C++ associative containers. | https://github.com/sparsehash/sparsehash | Other |
| Genetic variants annotation | |  |  |
| VEP | VEP determines the effect of your variants (SNPs, insertions, deletions, CNVs or structural variants) on genes, transcripts, and protein sequence, as well as regulatory regions. | http://www.ensembl.org/info/docs/tools/vep/index.html | Transcriptome |
| ANNOVAR | An efficient software tool to utilize update-to-date information to functionally annotate genetic variants detected from diverse genomes. | http://annovar.openbioinformatics.org/en/latest/ | Genome, Transcriptome |
| HAPSEG | The HAPSEG module takes single nucleotide polymorphism (SNP) microarray data and outputs copy number data segmented by haplotype. The output data is suitable for use as input data for the ABSOLUTE module. | http://software.broadinstitute.org/cancer/software/genepattern/modules/docs/HAPSEG/1 | Genome |
| SnpEff | Genomic variant annotations and functional effect prediction toolbox. | http://snpeff.sourceforge.net/index.html | Genome, Transcriptome |
| sequenza | A novel set of tools providing a fast python script to genotype cancer samples, and an R package to estimate cancer cellularity, ploidy, and genome wide copy number profile and infer for mutated alleles. | http://www.cbs.dtu.dk/biotools/sequenza/ | Genome |
| vcfanno | Annotate your VCF with any number of INFO fields from any number of VCFs or BED files. | https://github.com/brentp/vcfanno | Genome, Transcriptome |
| Oncotator | A web application for annotating human genomic point mutations and indels with data relevant to cancer researchers. | http://portals.broadinstitute.org/oncotator/ | Genome, Transcriptome |
| GIGGLE | GIGGLE is a genomics search engine that identifies and ranks the significance of genomic loci shared between query features and thousands of genome interval files. | https://github.com/ryanlayer/giggle | Genome, Transcriptome |
| SUBREAD | The Subread software package is a tool kit for processing next- generation sequencing data. It includes Subread aligner, Subjunc exon-exon junction detector and feature Counts read summarization program. Subread aligner can be used to align both gDNA-seq and RNA-seq reads. Subjunc aligner was specified designed for the detection of exon-exon junction. For the mapping of RNA-seq reads, Subread performs local alignments and Subjunc performs global alignments. | http://subread.sourceforge.net/ | Genome, Transcriptome |
| MutSig | MutSig is a package of tools for analyzing mutation data. It operates on a cohort of patients and identifies mutations, genes, and other genomic elements predicted to be driver candidates. | https://software.broadinstitute.org/cancer/cga/mutsig | Genome, Transcriptome |
| bedtools | The swiss army knife for genome arithmetic. | https://github.com/arq5x/bedtools2 | Genome, Transcriptome |
| OncodriveCLUST | Exploiting the positional clustering of somatic mutations to identify cancer genes. | http://bg.upf.edu/group/projects/oncodrive-clust.php | Genome, Transcriptome |
| Detection of SNVs, INDELs and SVs | |  |  |
| breakdancer | SV detection from paired end reads mapping. | https://github.com/genome/breakdancer | Genome |
| CNVkit | Copy number variant detection from targeted DNA sequencing. | https://github.com/etal/cnvkit | Genome |
| delly | Structural variant discovery by integrated paired-end and split-read analysis. | https://github.com/dellytools/delly | Genome |
| facets | Algorithm to implement Fraction and Copy number Estimate from Tumor/normal Sequencing. | https://github.com/mskcc/facets | Genome |
| freebayes | Bayesian haplotype-based genetic polymorphism discovery and genotyping. | https://github.com/ekg/freebayes | Genome |
| JAFFA | A multi-step pipeline that takes either raw RNA-Seq reads, or pre-assembled transcripts, then searches for gene fusions. | https://github.com/Oshlack/JAFFA | Transcriptome |
| lofreq | Sensitive variant calling from sequencing data. | http://csb5.github.io/lofreq/ | Genome, Transcriptome |
| manta | Structural variant and indel caller using mapped sequencing data. | https://github.com/Illumina/manta | Genome |
| Picky | Structural variants pipeline for long reads. | https://github.com/TheJacksonLaboratory/Picky | Genome |
| PRADA | Massively parallel sequencing of cDNA reverse transcribed from RNA (RNASeq) provides an accurate estimate of the quantity and composition of mRNAs. | https://sourceforge.net/projects/prada/ | Transcriptome |
| pindel | Detect breakpoints of large deletions, medium sized insertions, inversions, tandem duplications and other structural variants at single-based resolution from next-generation sequence data. | https://github.com/genome/pindel | Genome |
| SomaticSniper | A tool to call somatic single nucleotide variants. | https://github.com/genome/somatic-sniper | Genome, Transcriptome |
| speedseq | A flexible framework for rapid genome analysis and interpretation. | https://github.com/hall-lab/speedseq | Genome |
| svaba | Structural variation and indel detection by local assembly. | https://github.com/walaj/svaba | Genome |
| VarScan2 | Variant detection in massively parallel sequencing data. | http://dkoboldt.github.io/varscan/ | Genome, Transcriptome |
| RADIA | RADIA identifies RNA and DNA variants in BAM files. RADIA is typically run on 3 BAM files consisting of the Normal DNA, Tumor DNA and Tumor RNA. | https://github.com/aradenbaugh/radia/ | Genome, Transcriptome |
| RUM | An alignment, junction calling, and feature quantification pipeline specifically designed for Illumina RNA-Seq data. | https://github.com/itmat/rum | Transcriptome |
| FusionCatcher | Finder of Somatic Fusion Genes in RNA-seq data. | https://github.com/ndaniel/fusioncatcher | Transcriptome |
| GATK | Variant Discovery in High-Throughput Sequencing Data. | https://software.broadinstitute.org/gatk/ | Genome, Transcriptome |
| GRIDSS | The Genomic Rearrangement Identification Software Suite. | https://github.com/PapenfussLab/gridss | Genome |
| MuTect | Reliable and accurate identification of somatic point mutations in high-throughput sequencing data of cancer genomes. | http://archive.broadinstitute.org/cancer/cga/mutect/ | Genome, Transcriptome |
| Strelka | Accurate somatic small-variant calling from sequenced tumor–normal sample pairs. | ftp://strelka@ftp.illumina.com/ | Genome |
| svtoolkit | A suite of tools for discovering and genotyping structural variations using sequencing data. | http://software.broadinstitute.org/software/genomestrip/ | Genome |
| VADIR | An integrated approach to variant detection in RNA. | https://omictools.com/variant-detection-in-rna-tool | Transcriptome |
| TVC | Torrent Variant Caller. | https://github.com/iontorrent/TS | Genome, Transcriptome |
| CNVnator | A tool for CNV discovery and genotyping from depth-of-coverage by mapped reads. | https://github.com/abyzovlab/CNVnator | Genome |
| FREEC | Copy number and genotype annotation from whole genome and whole exome sequencing data. | https://github.com/BoevaLab/FREEC | Genome |
| REDItools | REDItools are python scripts developed with the aim to study RNA editing at genomic scale by next generation sequencing data. | https://sourceforge.net/projects/reditools/ | Transcriptome |
| Atlas2 | Atlas2 is a next-generation sequencing suite of variant analysis tools specializing in the separation of true SNPs and insertions and deletions (indels) from sequencing and mapping errors in Whole Exome Capture Sequencing (WECS) data. | https://sourceforge.net/projects/atlas2/ | Genome |
| SingleSplice | Algorithm for detecting alternative splicing in a population of single cells. | https://github.com/jw156605/SingleSplice | Single Cell |
| scLVM | scLVM is a modelling framework for single-cell RNA-seq data that can be used to dissect the observed heterogeneity into different sources, thereby allowing for the correction of confounding sources of variation. | https://github.com/PMBio/scLVM | Single Cell |
| Meerkat | Identification of somatic rearrangements in cancer genomes. | http://compbio.med.harvard.edu/Meerkat/ | Genome, Transcriptome |
| Immunity-associated | |  |  |
| TraCeR | Reconstruction of T cell receptor sequences from single-cell RNAseq data. | https://github.com/teichlab/tracer | Single Cell |
| ROP | Discovering the source of all RNA-seq reads, including those originating from repeat sequences, recombinant B and T cell receptors, and microbial communities. | https://github.com/smangul1/rop | Transcriptome |
| Isoform analysis | |  |  |
| ISOP | ISOform-level expression Patterns in single-cell RNA-sequencing data. | https://github.com/nghiavtr/ISOP | Single Cell |
| Outrigger | Large-scale detection and calculation of alternative splicing. | https://github.com/YeoLab/outrigger | Transcriptome |
| BRIE | Bayesian Regression for Isoform Estimate. | https://github.com/huangyh09/brie | Transcriptome |
| Gene expression analysis | |  |  |
| PRADA | Massively parallel sequencing of cDNA reverse transcribed from RNA (RNASeq) provides an accurate estimate of the quantity and composition of mRNAs. | https://sourceforge.net/projects/prada/ | Transcriptome |
| RESM | A software package for estimating gene and isoform expression levels from RNA-Seq data. | https://github.com/deweylab/RSEM | Transcriptome |
| SUBREAD | The Subread software package is a tool kit for processing next- generation sequencing data. It includes Subread aligner, Subjunc exon-exon junction detector and feature Counts read summarization program. Subread aligner can be used to align both gDNA-seq and RNA-seq reads. Subjunc aligner was specified designed for the detection of exon-exon junction. For the mapping of RNA-seq reads, Subread performs local alignments and Subjunc performs global alignments. | http://subread.sourceforge.net/ | Genome, Transcriptome |
| HTSeq | A Python library to facilitate processing and analysis of data from high-throughput sequencing (HTS) experiments. | https://github.com/simon-anders/htseq | Genome, Transcriptome |
| MDSeq | Gene expression mean and variability analysis for RNA-seq counts. | https://github.com/zjdaye/MDSeq | Transcriptome |
| sleuth | Differential analysis of RNA-Seq. | https://github.com/pachterlab/sleuth | Transcriptome |
| Strawberry | A program for fast and accurate genome-guided transcripts reconstruction and quantification from paired-end RNA-seq. | https://github.com/ruolin/strawberry | Transcriptome |
| SCnorm | An R package for normalizing single-cell RNA-seq data. | http://www.biostat.wisc.edu/~kendzior/SCNORM/ | Single Cell |
| aRNApipe | A project-oriented pipeline for processing of RNA-seq data in high performance cluster environments. | https://github.com/HudsonAlpha/aRNAPipe | Transcriptome |
| CHIP-seq analysis | |  |  |
| MACS | Model-based Analysis of ChIP-Seq. | https://github.com/taoliu/MACS | CHIP-seq |
| small RNA analysis | |  |  |
| sRNAnalyzer | sRNAnalyzer is a flexible, modular pipeline for the analysis of small RNA sequencing data. | http://srnanalyzer.systemsbiology.net/ | Transcriptome |
| Single cell analysis | |  |  |
| ASAP | Automated Single-cell Analysis Pipeline. | https://github.com/DeplanckeLab/ASAP | Single Cell |
| f-scLVM | Scalable and versatile factor analysis for single-cell RNA-seq. | https://github.com/PMBio/f-scLVM | Single Cell |
| RaceID | Algorithm for the inference of cell types from single-cell RNA-seq data. | https://github.com/dgrun/RaceID | Single Cell |
| scLVM | scLVM is a modelling framework for single-cell RNA-seq data that can be used to dissect the observed heterogeneity into different sources, thereby allowing for the correction of confounding sources of variation. | https://github.com/PMBio/scLVM | Single Cell |
| RCA | An R package for robust clustering analysis of single cell RNA sequencing data. | https://github.com/GIS-SP-Group/RCA | Single Cell |
| SingleSplice | Algorithm for detecting alternative splicing in a population of single cells. | https://github.com/jw156605/SingleSplice | Single Cell |
| MIMOSCA | Multiple Input Multiple Output Single Cell Analysis. | https://github.com/asncd/MIMOSCA | Single Cell |
| TraCeR | Reconstruction of T cell receptor sequences from single-cell RNAseq data. | https://github.com/teichlab/tracer | Single Cell |
| seurat | R toolkit for single cell genomics. | https://github.com/satijalab/seurat | Single Cell |
| ZIFA | Zero-inflated dimensionality reduction algorithm for single-cell data. | https://github.com/epierson9/ZIFA | Single Cell |
| BEARscc | Bayesian ERCC Assessment of Robustness. | https://github.com/Miachol/bearscc | Single Cell |
| ISOP | ISOform-level expression Patterns in single-cell RNA-sequencing data. | https://github.com/nghiavtr/ISOP | Single Cell |
| BackSPIN | BackSPIN biclustering algorithm. | https://github.com/linnarsson-lab/BackSPIN | Transcriptome,  Single Cell |
| SCnorm | An R package for normalizing single-cell RNA-seq data. | http://www.biostat.wisc.edu/~kendzior/SCNORM/ | Single Cell |
| Network analysis | |  |  |
| MARINa | MARINA (Master Regulator Inference Algorithm) MAster Regulator INference algorithm (MARINa), designed to infer transcription factors (TFs) controlling the transition between the two phenotypes, A and B, and the maintenance of the latter phenotype. | http://wiki.c2b2.columbia.edu/workbench/index.php/MARINa | Genome, Transcriptome |
| PARADIGM | Combine multiple-omics data to learn the strength and sign of regulatory interactions. | http://paradigm.five3genomics.com/ | Genome, Transcriptome |
| interproscan | InterProScan is the software package that allows sequences (protein and nucleic) to be scanned against InterPro's signatures. Signatures are predictive models, provided by several different databases, that make up the InterPro consortium. | http://www.ebi.ac.uk/interpro | Genome, Transcriptome,Proteome |
| Visualization | |  |  |
| IGV | The Integrative Genomics Viewer (IGV) is a high-performance visualization tool for interactive exploration of large, integrated genomic datasets. It supports a wide variety of data types, including array-based and next-generation sequence data, and genomic annotations. | http://www.igv.org/ | Genome, Transcriptome, CHIP-seq |
| ImageJ | A public domain Java image processing program inspired by NIH Image for the Macintosh. | https://imagej.nih.gov/ij/download.html | Genome, Transcriptome,Proteome |
| igraph | A library for creating and manipulating graphs. | https://github.com/igraph/igraph | Other |
| jvarkit | Java utilities for Bioinformatics. | https://github.com/lindenb/jvarkit | Genome, Transcriptome |
| FastQC | A quality control tool for high throughput sequence data. | http://www.bioinformatics.babraham.ac.uk/projects/fastqc/ | Genome, Transcriptome |
| samstat | SAMStat displays various properties of next-generation sequencing reads stored in SAM/BAM format. | https://sourceforge.net/projects/samstat | Genome, Transcriptome |
| SnpEff | Genomic variant annotations and functional effect prediction toolbox. | http://snpeff.sourceforge.net/index.html | Genome, Transcriptome |
| svtoolkit | A suite of tools for discovering and genotyping structural variations using sequencing data. | http://software.broadinstitute.org/software/genomestrip/ | Genome |
| VEP | VEP determines the effect of your variants (SNPs, insertions, deletions, CNVs or structural variants) on genes, transcripts, and protein sequence, as well as regulatory regions. | http://www.ensembl.org/info/docs/tools/vep/index.html | Transcriptome |
| BEARscc | Bayesian ERCC Assessment of Robustness. | https://github.com/Miachol/bearscc | Single Cell |
| root | A modular scientific software framework. It provides all the functionalities needed to deal with big data processing, statistical analysis, visualization and storage. | https://root.cern.ch/ | Other |
| System dependence | |  |  |
| R | A free software environment for statistical computing and graphics. | https://www.r-project.org/ | Other |
| MINICONDA2 | Conda image based on Python 2. | https://conda.io/miniconda.html | Other |
| MINICONDA3 | Conda image based on Python 3. | https://conda.io/miniconda.html | Other |
| PCRE | The PCRE library is a set of functions that implement regular expression pattern matching using the same syntax and semantics as Perl 5. | http://www.pcre.org/ | Other |
| pigz | A parallel implementation of gzip for modern multi-processor, multi-core machines. | http://zlib.net/pigz/ | Other |
| SQLite | SQLite is a self-contained, high-reliability, embedded, full-featured, public-domain, SQL database engine. | https://www.sqlite.org/index.html | Other |
| XZ | XZ Utils is free general-purpose data compression software with a high compression ratio. | https://tukaani.org/xz/ | Other |
| ZLIB | A compression library. | http://www.zlib.net/ | Other |
| LZO | Data compression. | http://www.oberhumer.com/products/lzo-professional/ | Other |
| LZOP | A file compressor. | http://www.lzop.org/ | Other |
| curl | Command line tool and library for transferring data with URLs. | https://curl.haxx.se/ | Other |
| htslib | HTSlib is an implementation of a unified C library for accessing common file formats, such as SAM, CRAM and VCF, used for high-throughput sequencing data, and is the core library used by samtools and bcftools. | https://github.com/samtools/htslib | Genome, Transcriptome |
| bzip2 | High-quality data compressor. | http://www.bzip.org/ | Genome, Transcriptome |
| pxz | Parallel LZMA compressor using liblzma. | https://github.com/jnovy/pxz | Other |
| libgtextutils | Gordon's Text utils Library. | https://github.com/agordon/libgtextutils | Other |
| ARMADILLO | C++ library for linear algebra & scientific computing. | http://arma.sourceforge.net/ | Other |
| sparsehash | C++ associative containers. | https://github.com/sparsehash/sparsehash | Other |
| vcflib | A simple C++ library for parsing and manipulating VCF files. | https://github.com/vcflib/vcflib | Genome, Transcriptome |

a. Partial description were extracted from online web sites of databases and the third party tool.
