# Supplementary material for: BioInstaller: a comprehensive R package to construct interactive and reproducible biological data analysis applications based on the R platform
Source: PeerJ. 2018 Oct 31;6:e5853. doi: 10.7717/peerj.5853 (PMC6215441; doi:10.7717/peerj.5853)
Supplement: Supplemental Information 2 [file peerj-06-5853-s002.docx]

| Database Name | Description^a^ | URL | Records |
| --- | --- | --- | --- |
| Variant-level |  |  |  |
| Allele frequency |  |  |  |
| annovar_brvar | A allele frequency database of 1,285 cases B-ALL RNA-seq data | http://bioinfo.rjh.com.cn/download/annovarR/humandb/ | 1 |
| annovar_normal_pool | A allele frequency database of leukemia patients control samples. | http://bioinfo.rjh.com.cn/download/annovarR/humandb/ | 1 |
| annovar_avsnp | dbSNP (http://www.bioinfo.org.cn/relative/dbSNP%20Home%20Page.htm) with allelic splitting and left-normalization | http://annovar.openbioinformatics.org/en/latest/user-guide/download/ | 1 |
| annovar_snp | dbSNP (http://www.bioinfo.org.cn/relative/dbSNP%20Home%20Page.htm) with ANNOVAR index files | http://annovar.openbioinformatics.org/en/latest/user-guide/download/ | 1 |
| annovar_cosmic | COSMIC (https://cancer.sanger.ac.uk/cosmic/) database | http://annovar.openbioinformatics.org/en/latest/user-guide/download/ | 1 |
| annovar_icgc21 | International Cancer Genome Consortium version 21 (https://icgc.org/) | http://annovar.openbioinformatics.org/en/latest/user-guide/download/ | 1 |
| annovar_cg | Alternative allele frequency in unrelated human subjects sequenced by Complete Genomics | http://annovar.openbioinformatics.org/en/latest/user-guide/download/ | 1 |
| annovar_esp6500siv2 | Alternative allele frequency in European American subjects in the NHLBI-ESP project with 6500 exomes, including the indel calls and the chrY calls | http://annovar.openbioinformatics.org/en/latest/user-guide/download/ | 1 |
| annovar_exac03 | ExAC 65000 exome allele frequency data for ALL, AFR (African), AMR (Admixed American), EAS (East Asian), FIN (Finnish), NFE (Non-finnish European), OTH (other), SAS (South Asian)). version 0.3. Left normalization done. | http://annovar.openbioinformatics.org/en/latest/user-guide/download/ | 1 |
| annovar_gme | Great Middle East (https://www.ncbi.nlm.nih.gov/pubmed/27428751) allele frequency including NWA (northwest Africa), NEA (northeast Africa), AP (Arabian peninsula), Israel, SD (Syrian desert), TP (Turkish peninsula) and CA (Central Asia) | http://annovar.openbioinformatics.org/en/latest/user-guide/download/ | 1 |
| annovar_gnomad | The Genome Aggregation Database (gnomAD, <http://gnomad.broadinstitute.org/>) is a resource developed by an international coalition of investigators, with the goal of aggregating and harmonizing both exome and genome sequencing data from a wide variety of large-scale sequencing projects, and making summary data available for the wider scientific community. | http://annovar.openbioinformatics.org/en/latest/user-guide/download/ | 1 |
| annovar_hrcr1 | 40 million variants from 32K samples in haplotype reference consortium | http://annovar.openbioinformatics.org/en/latest/user-guide/download/ | 1 |
| annovar_tmcsnpdb | An Indian germline variant database derived from whole exome sequences (https://academic.oup.com/database/article/doi/10.1093/database/baw104/2630486). | http://annovar.openbioinformatics.org/en/latest/user-guide/download/ | 1 |
| annovar_abraom | 2.3 million Brazilian genomic variants (https://www.ncbi.nlm.nih.gov/pubmed/28332257) | http://annovar.openbioinformatics.org/en/latest/user-guide/download/ | 1 |
| annovar_kaviar | 170 million Known VARiants from 13K genomes and 64K exomes in 34 projects | http://annovar.openbioinformatics.org/en/latest/user-guide/download/ | 1 |
| annovar_nci60 | NCI-60 human tumor cell line panel exome sequencing allele frequency data | http://annovar.openbioinformatics.org/en/latest/user-guide/download/ | 1 |
| annovar_popfreq | A database containing the maximum allele frequency from 1000G, ESP6500, ExAC and CG46 | http://annovar.openbioinformatics.org/en/latest/user-guide/download/ | 1 |
| Variants Effect prediction |  |  |  |
| annovar_seeqtl | seeQTL (https://seeqtl.org/): A searchable human eQTL browser and database | http://bioinfo.rjh.com.cn/download/annovarR/humandb/ | 1 |
| annovar_radar | RADAR: a rigorously annotated database of A-to-I RNA editing (http://rnaedit.com/) | http://bioinfo.rjh.com.cn/download/annovarR/humandb/ | 1 |
| annovar_ rediportal | REDIportal: An ATLAS of A-to-I RNA editing events in human and other organisms (http://srv00.recas.ba.infn.it/atlas/) | http://bioinfo.rjh.com.cn/download/annovarR/humandb/ | 1 |
| annovar_darned | DARNED: Database of RNA-editing in flies, mice and humans (https://darned.ucc.ie/) | http://bioinfo.rjh.com.cn/download/annovarR/humandb/ | 1 |
| annovar_dhs_gene_connectivity | Genomic coordinates of all promoter DHSs and distal, non-promoter DHSs within +-500 kb correlated with them at threshold 0.7 (https://www.nature.com/articles/nature11232) | http://bioinfo.rjh.com.cn/download/annovarR/humandb/ | 1 |
| DiseaseEnhancer | DiseaseEnhancer provides a comprehensive map of manually curated disease-associated enhancers, which includes 1059 disease-associated enhancers in 167 human diseases, involving 896 unique enhancer-gene interactions | http://biocc.hrbmu.edu.cn/DiseaseEnhancer/ | 1 |
| exsnp | ExSNP: An Integrated eQTL Database | http://www.exsnp.org | 1 |
| PancanQTL | PancanQTL: systematic identification of cis-eQTLs and trans-eQTLs in 33 cancer types. | http://bioinfo.life.hust.edu.cn/PancanQTL/ | 1 |
| rSNPBase3.0 | SNP-related regulatory elements, element-genn pairs & SNP-based regulatory network | http://rsnp3.psych.ac.cn/index.do | 1 |
| RDDpred | RDDpred: A condition-specific RNA-editing prediction model from RNA-seq data | http://epigenomics.snu.ac.kr/RDDpred/ | 1 |
| rVarBase | rVarBase: an updated database for regulatory features of human variants. | http://rv.psych.ac.cn | 1 |
| RBP-Var2 | RBP-Var2: A platform for exploring functional variants involved in post-transcriptional regulation mediated by RNA-binding proteins | http://www.rbp-var.biols.ac.cn/ | 1 |
| ReMap | ReMap: An integrative ChIP-seq analysis of regulatory regions | http://tagc.univ-mrs.fr/remap/ | 2 |
| annovar_gdi_score | GDI score: the gene damage index (GDI) is describing the accumulated mutational damage for each human gene in the general population, and shows that highly mutated/damaged genes are unlikely to be disease-causing and yet they generate a big proportion of false positive variants harbored in such genes. Therefore removing high GDI genes is a very effective way to remove confidently false positives from WES/WGS data. (http://www.pnas.org/content/early/2015/10/14/1518646112.abstract) | http://annovar.openbioinformatics.org/en/latest/user-guide/download/ | 1 |
| annovar_avsift | Whole-exome SIFT scores for non-synonymous variants. (https://www.ncbi.nlm.nih.gov/pubmed/19561590) | http://annovar.openbioinformatics.org/en/latest/user-guide/download/ | 1 |
| annovar_cadd | CADD is a tool for scoring the deleteriousness of single nucleotide variants as well as insertion/deletions variants in the human genome. (http://cadd.gs.washington.edu/) | http://annovar.openbioinformatics.org/en/latest/user-guide/download/ | 1 |
| annovar_dbnsfp | whole-exome SIFT, PolyPhen2 HDIV, PolyPhen2 HVAR, LRT, MutationTaster, MutationAssessor, FATHMM, PROVEAN, MetaSVM, MetaLR, VEST, M-CAP, CADD, GERP++, DANN, fathmm-MKL, Eigen, GenoCanyon, fitCons, PhyloP and SiPhy scores from dbNSFP (http://varianttools.sourceforge.net/Annotation/DbNSFP) | http://annovar.openbioinformatics.org/en/latest/user-guide/download/ | 1 |
| annovar_loftool_scores | LoFtool score: gene loss-of-function score percentiles. The smaller the percentile, the most intolerant is the gene to functional variation. Manuscript in preparation (please contact Dr. Joao Fadista - joao.fadista@med.lu.se) | http://annovar.openbioinformatics.org/en/latest/user-guide/download/ | 1 |
| annovar_rvis_esv_score | RVIS-ESV score: RVIS score measures genetic intolerance of genes to functional mutations, as described in Petrovski et al (http://journals.plos.org/plosgenetics/article?id=10.1371/journal.pgen.1003709). | http://www.openbioinformatics.org/annovar/download/ | 1 |
| annovar_fathmm | Whole-genome FATHMM_coding and FATHMM_noncoding scores (http://fathmm.biocompute.org.uk/). | http://annovar.openbioinformatics.org/en/latest/user-guide/download/ | 1 |
| annovar_gerp | Conserved genomic regions by GERP++ (http://mendel.stanford.edu/SidowLab/downloads/gerp/) | http://annovar.openbioinformatics.org/en/latest/user-guide/download/ | 1 |
| annovar_ljb26_all | whole-exome SIFT, PolyPhen2 HDIV, PolyPhen2 HVAR, LRT, MutationTaster, MutationAssessor, FATHMM, MetaSVM, MetaLR, VEST, CADD, GERP++, PhyloP and SiPhy scores from dbNSFP version 2.6 | http://annovar.openbioinformatics.org/en/latest/user-guide/download/ | 1 |
| annovar_dbscsnv | dbscSNV version 1.1 for splice site prediction by AdaBoost and Random Forest | http://annovar.openbioinformatics.org/en/latest/user-guide/download/ | 1 |
| annovar_eigen | Whole-genome Eigen scores (http://www.ncbi.nlm.nih.gov/pubmed/26727659) | http://annovar.openbioinformatics.org/en/latest/user-guide/download/ | 1 |
| annovar_gwava | Whole genome GWAVA_region_score and GWAVA_tss_score (http://www.nature.com/nmeth/journal/v11/n3/abs/nmeth.2832.html). | http://annovar.openbioinformatics.org/en/latest/user-guide/download/ | 1 |
| annovar_mcap | M-CAP scores for non-synonymous variants (http://bejerano.stanford.edu/mcap/index.html). | http://annovar.openbioinformatics.org/en/latest/user-guide/download/ | 1 |
| annovar_mitimpact | Pathogenicity predictions of human mitochondrial missense variants (http://www.ncbi.nlm.nih.gov/m/pubmed/25516408/). | http://annovar.openbioinformatics.org/en/latest/user-guide/download/ | 1 |
| annovar_regsnpintron | Prioritize the disease-causing probability of intronic SNVs (http://clark.compbio.iupui.edu/regsnp_intron_web/). | http://annovar.openbioinformatics.org/en/latest/user-guide/download/ | 1 |
| annovar_revel | REVEL scores for non-synonymous variants (https://sites.google.com/site/revelgenomics/about). | http://annovar.openbioinformatics.org/en/latest/user-guide/download/ | 1 |
| Disease-related |  |  |  |
| annovar_cancer_hotspots | http://cancerhotspots.org/#/home | http://bioinfo.rjh.com.cn/download/annovarR/humandb/ | 1 |
| annovar_docm | DoCM, the Database of Curated Mutations, is a highly curated database of known, disease-causing mutations that provides easily explorable variant lists with direct links to source citations for easy verification. http://www.docm.info/ | http://bioinfo.rjh.com.cn/download/annovarR/humandb/ | 1 |
| denovo-db | denovo-db (current release v.1.6) is a collection of germline de novo variants identified in the human genome. de novo variants are those present in children but not their parents. | http://denovo-db.gs.washington.edu/denovo-db | 1 |
| SEECancer | The SEECancer database presents the comprehensive cancer evolutionary stage-specific somatic events (including early-specific, late-specific, relapse-specific, metastasis-specific, drug-resistant and drug-induced genomic events) and their temporal orders. All records in SEECancer are manually verified. Through processing more than 10,000 published articles, the current version of SEECancer contains 1231 evolutionary stage-specific genomic events and 5772 temporal orders between different types of somatic changes across 82 human cancers, referring to 23 tissue origins (https://academic.oup.com/nar/advance-article/doi/10.1093/nar/gkx964/4561648). | http://biocc.hrbmu.edu.cn/SEECancer/ | 1 |
| annovar_intervar | InterVar: clinical interpretation of missense variants (http://wintervar.wglab.org/). | http://annovar.openbioinformatics.org/en/latest/user-guide/download/ | 1 |
| annovar_clinvar | ClinVar database with variant clinical significance (unknown, untested, non-pathogenic, probable-non-pathogenic, probable-pathogenic, pathogenic, drug-response, histocompatibility, other) and variant disease name (https://www.ncbi.nlm.nih.gov/clinvar/). | http://annovar.openbioinformatics.org/en/latest/user-guide/download/ | 1 |
| Gene-level |  |  |  |
| Basic information |  |  |  |
| annovar_gtex_eqtl_egenes | eGene based on permutations. (https://www.gtexportal.org). | http://bioinfo.rjh.com.cn/download/annovarR/humandb/ | 1 |
| annovar_gtex_eqtl_pairs | Significant variant-gene associations based on permutations. (https://www.gtexportal.org) | http://bioinfo.rjh.com.cn/download/annovarR/humandb/ | 1 |
| annovar_hgnc | <ftp://ftp.ebi.ac.uk/pub/databases/genenames/new/tsv/hgnc_complete_set.txt>. HGNC is responsible for approving unique symbols and names for human loci, including protein coding genes, ncRNA genes and pseudogenes, to allow unambiguous scientific communication. | http://bioinfo.rjh.com.cn/download/annovarR/humandb/ | 1 |
| annovar_ensgene | FASTA sequences for all annotated transcripts in ENSEMBL Gene | http://annovar.openbioinformatics.org/en/latest/user-guide/download/ | 1 |
| annovar_refgene | FASTA sequences for all annotated transcripts in RefSeq Gene | http://annovar.openbioinformatics.org/en/latest/user-guide/download/ | 1 |
| annovar_knowngene | FASTA sequences for all annotated transcripts in UCSC Known Gene | http://annovar.openbioinformatics.org/en/latest/user-guide/download/ | 1 |
| FANTOM | FANTOM is an international research consortium established by Dr. Hayashizaki and his colleagues in 2000 to assign functional annotations to the full-length cDNAs that were collected during the Mouse Encyclopedia Project at RIKEN. | http://fantom.gsc.riken.jp | 6 |
| Expression Atlas | Expression Atlas is an open science resource that gives users a powerful way to find information about gene and protein expression across species and biological conditions such as different tissues, cell types, developmental stages and diseases among others. | https://www.ebi.ac.uk/gxa/home/ | 1 |
| Gene function |  |  |  |
| annovar_epi_genes | A epigenetic genes classification database collected from this paper (https://www.ncbi.nlm.nih.gov/pubmed/?term=24710217). | http://bioinfo.rjh.com.cn/download/annovarR/humandb/ | 1 |
| EggNOG | A database of orthologous groups and functional annotation | http://eggnogdb.embl.de/#/app/downloads | 1 |
| MeDReaders | MeDReaders: A database for transcription factors that bind to methylated DNA | http://medreader.org/download | 1 |
| Disease-related |  |  |  |
| annovar_civic_gene_summaries | Clinical annotation database CIViC | https://civic.genome.wustl.edu/downloads/ | 1 |
| annovar_disgenet | The DisGeNET database integrates human gene-disease associations (GDAs) from various expert curated databases and text-mining derived associations including Mendelian, complex and environmental diseases. http://www.disgenet.org/web/DisGeNET/menu/downloads | http://bioinfo.rjh.com.cn/download/annovarR/humandb/ | 1 |
| annovar_omim_genemap2 | OMIM genemap2 txt file, download from http://www.omim.org/ | http://bioinfo.rjh.com.cn/download/annovarR/humandb/ | 1 |
| HPO | The Human Phenotype Ontology (HPO) provides a standardized vocabulary of phenotypic abnormalities encountered in human disease. Each term in the HPO describes a phenotypic abnormality, such as Atrial septal defect (https://hpo.jax.org/app/). This monthly job generates the data that is based on HPO-obo-file and the annotation-file. | http://compbio.charite.de/jenkins/job/hpo.annotations.monthly | 1 |
| annovar_tall_somatic_genes | Collected from recently published papers, 1) Recurrent SPI1 (PU.1) fusions in high-risk pediatric T cell acute lymphoblastic leukemia 2) The genomic landscape of pediatric and young adult T-lineage acute lymphoblastic leukemia 3) Identification of fusion genes and characterization of transcriptome features in T-cell acute lymphoblastic leukemia | http://bioinfo.rjh.com.cn/download/annovarR/humandb/ | 1 |
| annovar_intogen | IntOGen-mutations platform (http://www.intogen.org/mutations/) summarizes somatic mutations, genes and pathways involved in tumorigenesis. It identifies and visualizes cancer drivers, analyzing 4,623 exomes from 13 cancer sites. It provides support to cancer researchers, aids the identification of drivers across tumor cohorts and helps rank mutations for better clinical decision-making. | http://bioinfo.rjh.com.cn/download/annovarR/humandb/ | 1 |
| TumorFusions | TumorFusions: a data portal that catalogues 20 731 gene fusions detected in 9966 well characterized cancer samples and 648 normal specimens from The Cancer Genome Atlas (TCGA) | http://www.tumorfusions.org/ | 1 |
| Drug related |  |  |  |
| DGIdb | The Drug-Gene Interaction database (DGIdb) mines existing resources that generate hypotheses about how mutated genes might be targeted therapeutically or prioritized for drug development. It provides an interface for searching lists of genes against a compendium of drug-gene interactions and potentially ‘druggable’ genes. | http://dgidb.org/ | 1 |
| Drugbank | drugbank_all_full_database .xml.zip download from https://www.drugbank.ca | http://bioinfo.rjh.com.cn/download/bioinstaller/ | 1 |
| ECOdrug | The ECOdrug database contains information on the Evolutionary Conservation Of human Drug targets in over 600 eukaryotic species The interface allows users to identify human drug targets to 1000+ legacy drugs and explore integrated orthologue predictions for the drug targets, transparently showing the confidence in the predictions both across methods and taxonomic groups (<http://www.ecodrug.org/>). | http://bioinfo.rjh.com.cn/download/bioinstaller/ | 1 |
| superdrug2 | SuperDRUG2 database is a unique, one-stop resource for approved/marketed drugs, containing more than 4,500 active pharmaceutical ingredients. We annotated drugs with regulatory details, chemical structures (2D and 3D), dosage, biological targets, physicochemical properties, external identifiers, side-effects and pharmacokinetic data. Different search mechanisms allow navigation through the chemical space of approved drugs. (http://cheminfo.charite.de/superdrug2/) | http://bioinfo.rjh.com.cn/download/bioinstaller/ | 1 |
| Noncoding RNA related |  |  |  |
| AtCircDB | AtCircDB: a tissue-specific database for Arabidopsis circular RNAs. | http://genome.sdau.edu.cn/circRNA/index.php | 1 |
| circBase | circBase: a database and website, “circBase,” where merged and unified data sets of circRNAs and the evidence supporting their expression can be accessed, downloaded, and browsed within the genomic context | http://circrna.org/ | 1 |
| annovar_cscd | A database for cancer-specific circular RNAs | http://gb.whu.edu.cn/CSCD/ | 1 |
| CircNet | Utilized transcriptome sequencing datasets to systematically identify the expression of circRNAs (including known and newly identified ones by our pipeline) in 464 RNA-seq samples, and then constructed the CircNet database. | http://circnet.mbc.nctu.edu.tw/ | 1 |
| circRNADb | A comprehensive database for human circular RNAs with protein-coding annotations | http://202.195.183.4:8000/circrnadb/circRNADb.php | 1 |
| miRDB | An online database for miRNA target prediction and functional annotations. | http://mirdb.org/download/ | 1 |
| miRNEST | An integrative collection of animal, plant and virus microRNA data. | http://rhesus.amu.edu.pl/mirnest/copy/downloads/ | 1 |
| miRTarBase | Accumulated more than three hundred and sixty thousand miRNA-target interactions (MTIs), which are collected by manually surveying pertinent literature after NLP of the text systematically to filter research articles related to functional studies of miRNAs. | http://mirtarbase.mbc.nctu.edu.tw/cache/download/ | 1 |
| miRCancer | miRCancer provides comprehensive collection of microRNA (miRNA) expression profiles in various human cancers which are automatically extracted from published literatures in PubMed. It utilizes text mining techniques for information collection. Manual revision is applied after auto-extraction to provide 100% precision. | http://mircancer.ecu.edu/downloads/ | 1 |
| exoRBase | A repository of circular RNA (circRNA), long non-coding RNA (lncRNA) and messenger RNA (mRNA) derived from RNA-seq data analyses of human blood exosomes. | http://www.exorbase.org/exoRBase/toIndex | 1 |
| LNCediting | A comprehensive resource for the functional prediction of RNA editing in long noncoding RNAs (lncRNAs) | http://bioinfo.life.hust.edu.cn/LNCediting | 1 |
| LNCipedia | A public database for long non-coding RNA (lncRNA) sequence and annotation. The current release contains 127,802 transcripts and 56,946 genes. | https://lncipedia.org/downloads/ | 1 |
| MSDD | MSDD: a manually curated database of experimentally supported associations among miRNAs, SNPs and human diseases | http://www.bio-bigdata.com/msdd/ | 1 |
| MNDR | MNDR v2.0 include (1) over 220-fold ncRNA-disease associations enhancement than previous version (including lncRNA, miRNA, piRNA, snoRNA and more than 1,400 diseases); (2) integrating experimental and prediction evidence from 14 resources and prediction algorithms for each ncRNA-disease association; (3) mapping disease name to the Disease Ontology and Medical Subject Headings (MeSH); (4) providing a confidence score for each ncRNA-disease association; and (5) an increase of species coverage to 6 mammals. | http://www.rna-society.org/mndr/ | 1 |
| sRNAnalyzer | A flexible, modular pipeline for the analysis of small RNA sequencing data (dependent databases: sRNA_DBs, MainDBs, NCBI_NonHuman). | http://srnanalyzer.systemsbiology.net/ | 1 |
| Reference genome |  |  |  |
| ucsc_reffa | Reference genome (hg38, hg19, mm9, mm10) from UCSC. | http://hgdownload.cse.ucsc.edu/goldenPath | 1 |
| ensemble_grch37_reffa | Reference genome from Ensemble (v75) | ftp://ftp.ensembl.org/pub | 1 |
| ensemble_grch38_reffa | Reference genome release from Ensemble (v76-93) | ftp://ftp.ensembl.org/pub | 1 |
| hisat2_reffa | Reference genome from Hisat2 host | ftp://ftp.ccb.jhu.edu/pub/infphilo/hisat2/data | 1 |
| jaffa_reffa | Reference genome from JAFFA | https://github.com/Oshlack/JAFFA/wiki/Download | 1 |
| fusioncatcher_reffa | Reference genome from fusioncatcher | https://github.com/ndaniel/fusioncatcher | 1 |
| gatk_bundle | Reference genome and other dependent files of GATK | https://software.broadinstitute.org/gatk/download/bundle | 1 |
| mutsig_reffa | Reference genome from MutSig | https://software.broadinstitute.org/cancer/cga/mutsig | 1 |
| rmats_reffa | Reference genome from rMATs | http://rnaseq-mats.sourceforge.net/download.html | 1 |
| Protein related |  |  |  |
| InterPro | InterPro provides functional analysis of proteins by classifying them into families and predicting domains and important sites. | http://www.ebi.ac.uk/interpro | 1 |
| InBio Map | A high coverage, high quality, convenient and transparent platform for investigating and visualizing protein-protein interactions. | https://www.intomics.com/inbio/map | 1 |
| proteinatlas | The Human Protein Atlas (HPA) is a Swedish-based program started in 2003 with the aim to map of all the human proteins in cells, tissues and organs using integration of various omics technologies, including antibody-based imaging, mass spectrometry-based proteomics, transcriptomics and systems biology. | https://www.proteinatlas.org/ | 1 |
| RBPDB | RBPDB, the database of RNA-binding protein specificities | http://rbpdb.ccbr.utoronto.ca/downloads/ | 1 |
| Others |  |  |  |
| annovar_varcards | VarCards: an integrated genetic and clinical database for coding variants in the human genome (http://varcards.biols.ac.cn/) | http://bioinfo.rjh.com.cn/download/annovarR/humandb/ | 1 |
| Oncotator | A web application for annotating human genomic point mutations and indels with data relevant to cancer researchers (dependent databases). | http://portals.broadinstitute.org/oncotator/ | 1 |
| SNiPA | SNiPA offers both functional annotations and linkage disequilibrium information for bi-allelic genomic variants (SNPs and SNVs) | http://snipa.helmholtz-muenchen.de/snipa3 | 1 |
| UCSC integrated | UCSC integrated annotation databases | http://hgdownload.cse.ucsc.edu/downloads.html#human | 4995 |
| APPRIS | Annotating alternative splice isoforms in vertebrate genomes | http://apprisws.bioinfo.cnio.es/pub/current_release/datafiles/homo_sapiens/ | 1 |
| BioSystems | BioSystems provides integrated access to biological systems and their component genes, proteins, and small molecules, as well as literature describing those biosystems and other related data throughout Entrez. | ftp://ftp.ncbi.nih.gov/pub/biosystems/ | 1 |
| BLAST integrated | BLAST tool dependent databases | ftp://ftp.ncbi.nlm.nih.gov/blast/db/ | 29 |
| funcoup | The most comprehensive databases for genome-wide functional association networks. | http://funcoup.sbc.su.se/search/ | 1 |

a. Partial description were extracted from online web sites of databases and the third party tool.
