# Supplementary material for: BioInstaller: a comprehensive R package to construct interactive and reproducible biological data analysis applications based on the R platform
Source: PeerJ. 2018 Oct 31;6:e5853. doi: 10.7717/peerj.5853 (PMC6215441; doi:10.7717/peerj.5853)
Supplement: Supplemental Information 3 — A set of files with file size range from 0.25 GB to 8 GB were used to test the performance of upload and download files in Shiny application. [file peerj-06-5853-s003.docx]

| Shiny application with  local network (200Mbps) | Upload | Download |
| --- | --- | --- |
| 0.25 GB | 9s, 8s, 9s | 9s, 9s, 8s |
| 0.5 GB | 15s, 16s, 16s | 14s, 15s, 14s |
| 0.75 GB | 23s, 25s, 22s | 21s, 21s, 22s |
| 1 GB | 35s, 36s, 31s | 28s, 29s, 28s |
| 2 GB | 58s, 60s, 62s | 60s, 58s, 57s |
| 4 GB | 123s, 113s, 119s | 120s, 118s, 112 |
| 8 GB | 264s, 251s, 254s | 229s, 225s, 226 |

R command ‘options(shiny.maxRequestSize = 30000 * 1024^2)’ was used to set the maximum limitation

(30GB) of uploading files via using Shiny application. ‘client_max_body_size 30G’ was used to set the maximum limitation

(30GB) of uploading files via using nginx reverse proxy
