# Supplementary material for: BioInstaller: a comprehensive R package to construct interactive and reproducible biological data analysis applications based on the R platform
Source: PeerJ. 2018 Oct 31;6:e5853. doi: 10.7717/peerj.5853 (PMC6215441; doi:10.7717/peerj.5853)
Supplement: Supplemental Information 4 — (A) GitHub APIs integrated in R function can access GitHub projects meta information, including branches, tags and releases versions. (B) The demo function shows how to get the versions of the sequence alignment tool GMAP. (C) The demo configuration item shows how to download and install miniconda2 using mirror URL. [file peerj-06-5853-s004.pdf]

A

```r
library("configr")
library("stringr")
library("RCurl")
github2versions <- function(github.url) {
  github.url <- str_replace(github.url, "http://|https://|git://", "")
  txt <- str_split(github.url, "/")[[1]]
  user <- txt[2]
  repo <- txt[3]
  h <- basicTextGatherer()
  myheader <- c(`User-Agent` = paste0("Mozilla/5.0 (iPhone; U; CPU iPhone OS 4_0_1 like ",
    "Mac OS X; ja-jp) AppleWebKit/532.9 (KHTML, like Gecko) Version/4.0.5 Mobile/8A306 ",
    "Safari/6531.22.7"),
    Accept = "text/html,application/xhtml+xml,application/xml;q=0.9,*/*;q=0.8",
    `Accept-Language` = "en-us", Connection = "keep-alive", `Accept-Charset` =
    "GB2312,utf-8;q=0.7,*;q=0.7")
  url <- sprintf(paste0("https://api.github.com/repos/%s/%s/tags?",
  "client_id=1d40ab6884d214ef6889&",
  "client_secret=23b818c2bad8e9f88dafd8a425613475362b326d"),  user, repo)
  txt <- getURL(url, headerfunction = h$update, httpheader = myheader)
  json <- tempfile()
  cat(txt, file = json, sep = "\n")
  return(read.config(file = json)$name)
}
```

B

```r
library("configr")
library("stringr")
library("rvest")
get.gmap.versions <- function() {
  urls <- c("http://research-pub.gene.com/gmap/src/")
  versions_final <- NULL
  for (url in urls) {
    web <- read_html(url, encoding = "UTF-8")
    files.table <- web %>% html_nodes("table") %>% .[[1]] %>% html_table()
    files <- files.table$Name
    versions <- str_extract(files, "gmap-.*tar.gz")
    versions <- versions[!is.na(versions)]
    versions <- str_extract(versions, "gmap-.*.tar")
    versions <- str_replace(versions, ".tar$", "")
    versions_final <- c(versions_final, versions)
  }
  return(versions_final)
}
```

C

```toml
[miniconda2]
decompress = false
url_all_download = false

[miniconda2.source_url]
windows = ["https://repo.continuum.io/miniconda/Miniconda2-{{version}}-Windows-x86_64.exe",
           "http://bioinfo.rjh.com.cn/download/miniconda/Miniconda2-{{version}}-Windows-x86_64.exe"]
mac = ["https://repo.continuum.io/miniconda/Miniconda2-{{version}}-MacOSX-x86_64.sh",
       "http://bioinfo.rjh.com.cn/download/miniconda/Miniconda2-{{version}}-MacOSX-x86_64.sh"]
linux = ["https://repo.continuum.io/miniconda/Miniconda2-{{version}}-Linux-x86_64.sh",
         "http://bioinfo.rjh.com.cn/download/miniconda/Miniconda2-{{version}}-Linux-x86_64.sh"]

[miniconda2.install]
linux = "sh Miniconda2-{{version}}-Linux-x86_64.sh"
mac = "sh Miniconda2-{{version}}-MacOSX-x86_64.sh"
```
