# Supplementary material for: BioInstaller: a comprehensive R package to construct interactive and reproducible biological data analysis applications based on the R platform
Source: PeerJ. 2018 Oct 31;6:e5853. doi: 10.7717/peerj.5853 (PMC6215441; doi:10.7717/peerj.5853)
Supplement: Supplemental Information 5 — (A) Overview of Shiny navigation bar tab items and the introduction module page. (B) Dashboard module page contains system monitor, task queue monitor, query system, and others. (C) Output table in dashboard module shows all files in ‘PATH’ directory’s environment variable. It also includes search and export functions. (D) UI of upload module contains extra fields, including file type, genome version, and description, to describe the information of the files. (E) Save button was used to save uploaded file and update the related database. (F) Preview box was used to view uploaded files before confirming uploading. [file peerj-06-5853-s005.pdf]

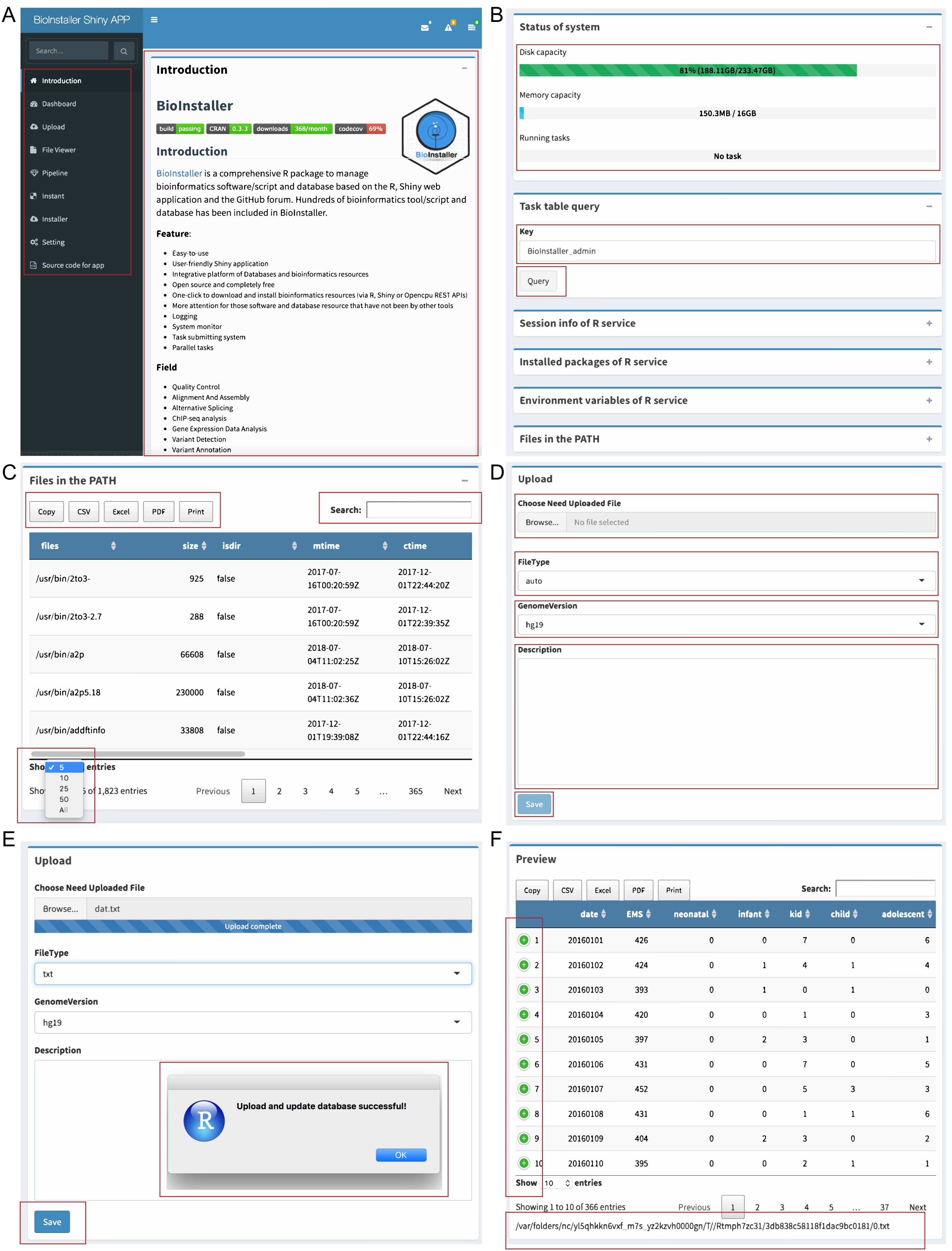
A
BioInstaller Shiny APP
Search...
Introduction
Dashboard
Upload
File Viewer
Pipeline
Instant
Installer
Setting
Source code for app
Introduction
BioInstaller
build passing
CRAN 0.3.3
downloads 368/month
codecov 69%
BioInstaller
Introduction
BioInstaller is a comprehensive R package to manage bioinformatics software/script and database based on the R, Shiny web application and the GitHub forum. Hundreds of bioinformatics tool/script and database has been included in BioInstaller.
Feature:
Easy-to-use
User-friendly Shiny application
Integrative platform of Databases and bioinformatics resources
Open source and completely free
One-click to download and install bioinformatics resources (via R, Shiny or Opencpu REST APIs)
More attention for those software and database resource that have not been by other tools
Logging
System monitor
Task submitting system
Parallel tasks
Field
Quality Control
Alignment And Assembly
Alternative Splicing
ChIP-seq analysis
Gene Expression Data Analysis
Variant Detection
Variant Annotation
B
Status of system
Disk capacity
81% (188.11GB/233.47GB)
Memory capacity
150.3MB / 16GB
Running tasks
No task
Task table query
Key
BioInstaller_admin
Query
Session info of R service
Installed packages of R service
Environment variables of R service
Files in the PATH
C
Files in the PATH
Copy CSV Excel PDF Print
Search:
files size isdir mtime ctime
/usr/bin/2to3- 925 false 2017-07-16T00:20:59Z 2017-12-01T22:44:20Z
/usr/bin/2to3-2.7 288 false 2017-07-16T00:20:59Z 2017-12-01T22:39:35Z
/usr/bin/a2p 66608 false 2018-07-04T11:02:25Z 2018-07-10T15:26:02Z
/usr/bin/a2p5.18 230000 false 2018-07-04T11:02:36Z 2018-07-10T15:26:02Z
/usr/bin/addftinfo 33808 false 2017-12-01T19:39:08Z 2017-12-01T22:44:16Z
Show 5 10 25 50 All entries
of 1,823 entries
Previous 1 2 3 4 5 … 365 Next
D
Upload
Choose Need Uploaded File
Browse... No file selected
FileType
auto
GenomeVersion
hg19
Description
Save
E
Upload
Choose Need Uploaded File
Browse... dat.txt
Upload complete
FileType
txt
GenomeVersion
hg19
Description
R
Upload and update database successful!
OK
Save
F
Preview
Copy CSV Excel PDF Print
Search:
date EMS neonatal infant kid child adolescent
1 20160101 426 0 0 7 0 6
2 20160102 424 0 1 4 1 4
3 20160103 393 0 1 0 1 0
4 20160104 420 0 0 1 0 3
5 20160105 397 0 2 3 0 1
6 20160106 431 0 0 7 0 5
7 20160107 452 0 0 5 3 3
8 20160108 431 0 0 1 1 6
9 20160109 404 0 2 3 0 2
10 20160110 395 0 0 2 1 1
Show 10 entries
Showing 1 to 10 of 366 entries
Previous 1 2 3 4 5 … 37 Next
/var/folders/nc/yl5qhkkn6vxf_m7s_yz2kzvh0000gn/T//Rtmph7zc31/3db838c58118f1dac9bc0181/0.txt
