# Supplementary material for: BioInstaller: a comprehensive R package to construct interactive and reproducible biological data analysis applications based on the R platform
Source: PeerJ. 2018 Oct 31;6:e5853. doi: 10.7717/peerj.5853 (PMC6215441; doi:10.7717/peerj.5853)
Supplement: Supplemental Information 6 — (A) The example of R session information of host running Shiny application. (B) Installed R package of the host running Shiny application. (C) Output table of all environment variables and its value. (D)(E)(F)Output table and text of installed items via BioInstaller, conda, and spack. [file peerj-06-5853-s006.pdf]

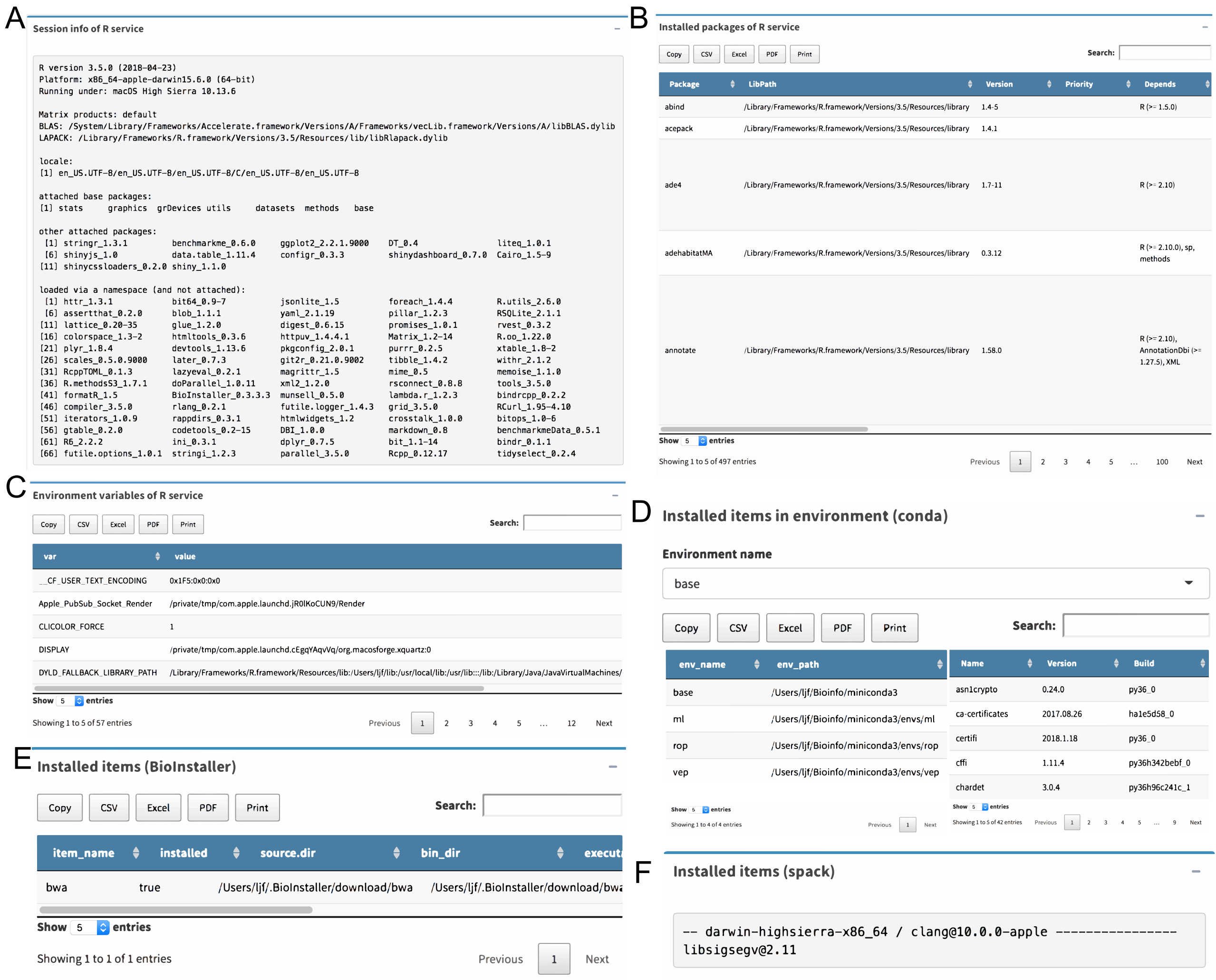

A
Session info of R service
R version 3.5.0 (2018-04-23)
Platform: x86_64-apple-darwin15.6.0 (64-bit)
Running under: macOS High Sierra 10.13.6
Matrix products: default
BLAS: /System/Library/Frameworks/Accelerate.framework/Versions/A/Frameworks/vecLib.framework/Versions/A/libBLAS.dylib
LAPACK: /Library/Frameworks/R.framework/Versions/3.5/Resources/lib/libRlapack.dylib
locale:
[1] en_US.UTF-8/en_US.UTF-8/en_US.UTF-8/C/en_US.UTF-8/en_US.UTF-8
attached base packages:
[1] stats graphics grDevices utils datasets methods base
other attached packages:
[1] stringr_1.3.1 benchmarkme_0.6.0 ggplot2_2.2.1.9000 DT_0.4 liteq_1.0.1
[6] shinyjs_1.0 data.table_1.11.4 configr_0.3.3 shinydashboard_0.7.0 Cairo_1.5-9
[11] shinycssloaders_0.2.0 shiny_1.1.0
loaded via a namespace (and not attached):
[1] httr_1.3.1 bit64_0.9-7 jsonlite_1.5 foreach_1.4.4 R.utils_2.6.0
[6] assertthat_0.2.0 blob_1.1.1 yaml_2.1.19 pillar_1.2.3 RSQLite_2.1.1
[11] lattice_0.20-35 glue_1.2.0 digest_0.6.15 promises_1.0.1 rvest_0.3.2
[16] colorspace_1.3-2 htmltools_0.3.6 httpuv_1.4.4.1 Matrix_1.2-14 R.oo_1.22.0
[21] plyr_1.8.4 devtools_1.13.6 pkgconfig_2.0.1 purrr_0.2.5 xtable_1.8-2
[26] scales_0.5.0.9000 later_0.7.3 git2r_0.21.0.9002 tibble_1.4.2 withr_2.1.2
[31] RcppTOML_0.1.3 lazyeval_0.2.1 magrittr_1.5 mime_0.5 memoise_1.1.0
[36] R.methodsS3_1.7.1 doParallel_1.0.11 xml2_1.2.0 rsconnect_0.8.8 tools_3.5.0
[41] formatR_1.5 BioInstaller_0.3.3.3 munsell_0.5.0 lambda.r_1.2.3 bindrcpp_0.2.2
[46] compiler_3.5.0 rlang_0.2.1 futile.logger_1.4.3 grid_3.5.0 RCurl_1.95-4.10
[51] iterators_1.0.9 rappdirs_0.3.1 htmlwidgets_1.2 crosstalk_1.0.0 bitops_1.0-6
[56] gtable_0.2.0 codetools_0.2-15 DBI_1.0.0 markdown_0.8 benchmarkmeData_0.5.1
[61] R6_2.2.2 ini_0.3.1 dplyr_0.7.5 bit_1.1-14 bindr_0.1.1
[66] futile.options_1.0.1 stringi_1.2.3 parallel_3.5.0 Rcpp_0.12.17 tidyselect_0.2.4
B
Installed packages of R service
Copy CSV Excel PDF Print
Search:
Package LibPath Version Priority Depends
abind /Library/Frameworks/R.framework/Versions/3.5/Resources/library 1.4-5 R (>= 1.5.0)
acepack /Library/Frameworks/R.framework/Versions/3.5/Resources/library 1.4.1
ade4 /Library/Frameworks/R.framework/Versions/3.5/Resources/library 1.7-11 R (>= 2.10)
adehabitatMA /Library/Frameworks/R.framework/Versions/3.5/Resources/library 0.3.12 R (>= 2.10.0), sp, methods
annotate /Library/Frameworks/R.framework/Versions/3.5/Resources/library 1.58.0 R (>= 2.10), AnnotationDbi (>= 1.27.5), XML
Show 5 entries
Showing 1 to 5 of 497 entries
Previous 1 2 3 4 5 … 100 Next
C
Environment variables of R service
Copy CSV Excel PDF Print
Search:
var value
__CF_USER_TEXT_ENCODING 0x1F5:0x0:0x0
Apple_PubSub_Socket_Render /private/tmp/com.apple.launchd.jR0lKoCUN9/Render
CLICOLOR_FORCE 1
DISPLAY /private/tmp/com.apple.launchd.cEgqYAqvVq/org.macosforge.xquartz:0
DYLD_FALLBACK_LIBRARY_PATH /Library/Frameworks/R.framework/Resources/lib:/Users/ljf/lib:/usr/local/lib:/usr/lib:::/lib:/Library/Java/JavaVirtualMachines/
Show 5 entries
Showing 1 to 5 of 57 entries
Previous 1 2 3 4 5 … 12 Next
D
Installed items in environment (conda)
Environment name
base
Copy CSV Excel PDF Print
Search:
env_name env_path
base /Users/ljf/Bioinfo/miniconda3
ml /Users/ljf/Bioinfo/miniconda3/envs/ml
rop /Users/ljf/Bioinfo/miniconda3/envs/rop
vep /Users/ljf/Bioinfo/miniconda3/envs/vep
Show 5 entries
Showing 1 to 4 of 4 entries
Previous 1 Next
Name Version Build
asn1crypto 0.24.0 py36_0
ca-certificates 2017.08.26 ha1e5d58_0
certifi 2018.1.18 py36_0
cffi 1.11.4 py36h342bebf_0
chardet 3.0.4 py36h96c241c_1
Show 5 entries
Showing 1 to 5 of 42 entries
Previous 1 2 3 4 5 … 9 Next
E
Installed items (BioInstaller)
Copy CSV Excel PDF Print
Search:
item_name installed source.dir bin_dir executi
bwa true /Users/ljf/.BioInstaller/download/bwa /Users/ljf/.BioInstaller/download/bwa
Show 5 entries
Showing 1 to 1 of 1 entries
Previous 1 Next
F
Installed items (spack)
-- darwin-highsierra-x86_64 / clang@10.0.0-apple ----------------
libsigsegv@2.11
