# Supplementary material for: BioInstaller: a comprehensive R package to construct interactive and reproducible biological data analysis applications based on the R platform
Source: PeerJ. 2018 Oct 31;6:e5853. doi: 10.7717/peerj.5853 (PMC6215441; doi:10.7717/peerj.5853)
Supplement: Supplemental Information 8 — (A) Rendered UI from the YAML format configuration file. (B) YAML editor allows users to add and modify variables used in the Shiny application. This can real-time update the UI of panel (A). [file peerj-06-5853-s008.pdf]

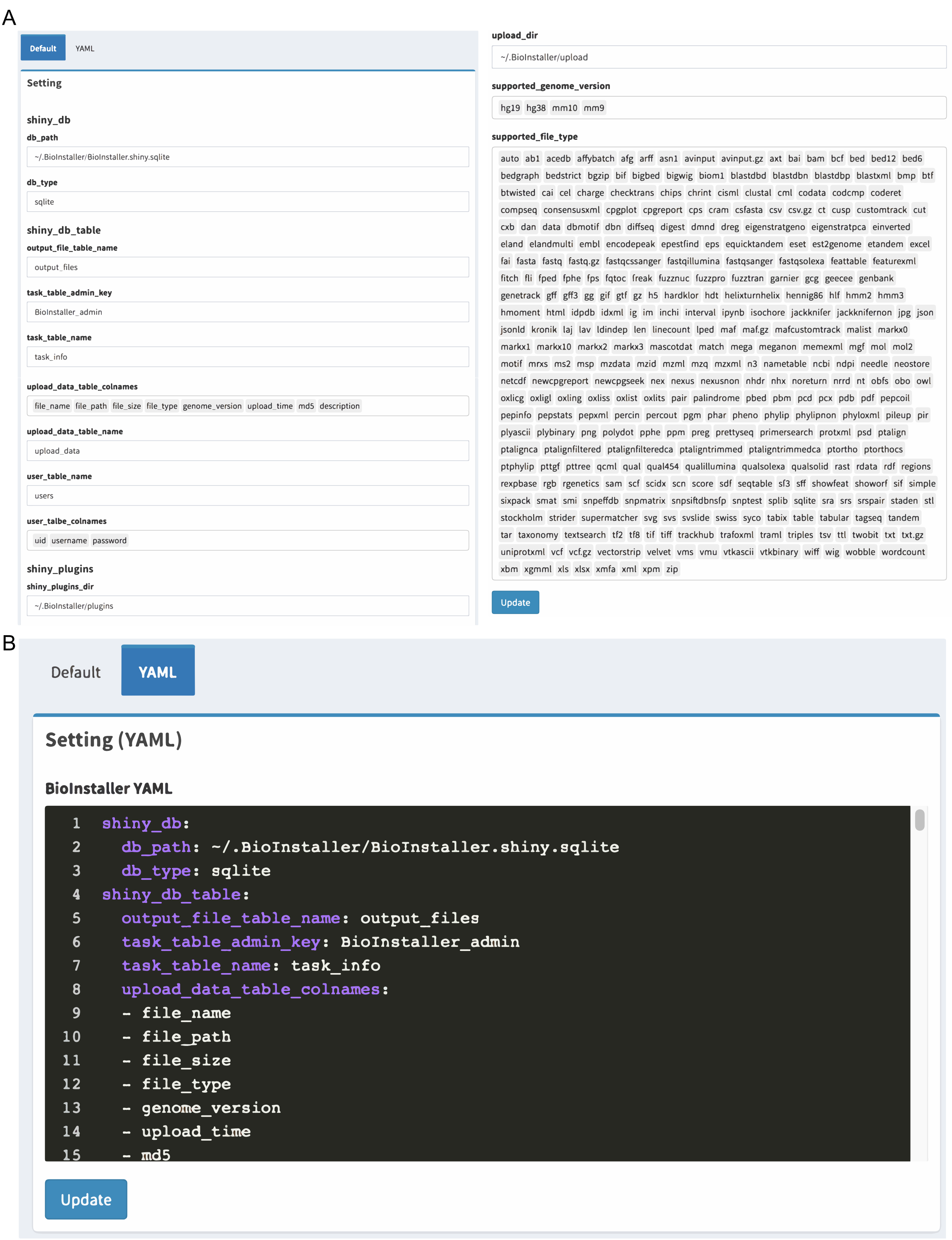
A
Default
YAML
Setting
shiny_db
db_path
~/.BioInstaller/BioInstaller.shiny.sqlite
db_type
sqlite
shiny_db_table
output_file_table_name
output_files
task_table_admin_key
BioInstaller_admin
task_table_name
task_info
upload_data_table_colnames
file_name file_path file_size file_type genome_version upload_time md5 description
upload_data_table_name
upload_data
user_table_name
users
user_talbe_colnames
uid username password
shiny_plugins
shiny_plugins_dir
~/.BioInstaller/plugins
upload_dir
~/.BioInstaller/upload
supported_genome_version
hg19 hg38 mm10 mm9
supported_file_type
auto ab1 acedb affybatch afg arff asn1 avinput avinput.gz axt bai bam bcf bed bed12 bed6 bedgraph bedstrict bgzip bif bigbed bigwig biom1 blastdbd blastdbn blastdbp blastxml bmp btf btwisted cai cel charge checktrans chips chrint cisml clustal cml codata codcmp coderet compseq consensusxml cpgplot cpgreport cps cram csfasta csv csv.gz ct cusp customtrack cut cxb dan data dbmotif dbn diffseq digest dmnd dreg eigenstratgeno eigenstratpca einverted eland elandmulti embl encodepeak epestfind eps equicktandem eset est2genome etandem excel fai fasta fastq fastq.gz fastqcssanger fastqillumina fastqsanger fastqsolexa feattable featurexml fitch fli fped fphe fps fqtoc freak fuzznuc fuzzpro fuzztran garnier gcg geecee genbank genetrack gff gff3 gg gif gtf gz h5 hardklor hdt helixturnhelix hennig86 hlf hmm2 hmm3 hmoment html idpdb idxml ig im inchi interval ipynb isochore jackknifer jackknifernon jpg json jsonld kronik laj lav ldindep len linecount lped maf maf.gz mafcustomtrack malist markx0 markx1 markx10 markx2 markx3 mascotdat match mega meganon memexml mgf mol mol2 motif mrxs ms2 msp mzdata mzid mzml mzq mzxml n3 nametable ncbi ndpi needle neostore netcdf newcpgreport newcpgseek nex nexus nexusnon nhdr nhx noreturn nrrd nt obfs obo owl oxlicg oxligl oxling oxliss oxlist oxlits pair palindrome pbed pbm pcd pcx pdb pdf pepcoil pepinfo pepstats pepxml percin percout pgm phar pheno phylip phylipnon phyloxml pileup pir plyascii plybinary png polydot pphe ppm preg prettyseq primersearch protxml psd ptalign ptaligna ptalignfiltered ptalignfilteredca ptaligntrimmed ptaligntrimmedca ptortho ptorthocs ptphylip pttgf pttree qcml qual qual454 qualillumina qualsolexa qualsolid rast rdata rdf regions rexpbase rgb rgenetics sam scf scidx scn score sdf seqtable sf3 sff showfeat showorf sif simple sixpack smat smi snpeffdb snpmatrix snpsiftdbnsfp snptest splib sqlite sra srs srspair staden stl stockholm strider supermatcher svg svs svslide swiss syco tabix table tabular tagseq tandem tar taxonomy textsearch tf2 tf8 tif tiff trackhub trafoxml traml triples tsv ttl twobit txt txt.gz uniprotxml vcf vcf.gz vectorstrip velvet vms vmu vtkascii vtkbinary wiff wig wobble wordcount xbm xgmml xls xlsx xmfa xml xpm zip
Update
B
Default
YAML
Setting (YAML)
BioInstaller YAML
1 shiny_db:
2 db_path: ~/.BioInstaller/BioInstaller.shiny.sqlite
3 db_type: sqlite
4 shiny_db_table:
5 output_file_table_name: output_files
6 task_table_admin_key: BioInstaller_admin
7 task_table_name: task_info
8 upload_data_table_colnames:
9 - file_name
10 - file_path
11 - file_size
12 - file_type
13 - genome_version
14 - upload_time
15 - md5
Update
