# Supplementary material for: BioInstaller: a comprehensive R package to construct interactive and reproducible biological data analysis applications based on the R platform
Source: PeerJ. 2018 Oct 31;6:e5853. doi: 10.7717/peerj.5853 (PMC6215441; doi:10.7717/peerj.5853)
Supplement: Supplemental Information 11 [file peerj-06-5853-s011.docx]

**One-stop to manage the environment of tools/scripts and databases**

We integrated multiple popular command line tools (e.g. conda and spack) with wrapped R functions and extra R functions to facilitate the deployment and management of the diverse analytical environment (**Figure 1**). The extra R functions are provided by BioInstaller and the R packages (e.g. <https://cran.r-project.org/package=configr> and https://cran.r-project.org/package=rvest). An integrated Shiny dashboard page is also available for users, which support one-stop to deploy massive bioinformatics resources (e.g. the reference genome, annotation databases, gene sets, etc.) and to export the installed or downloaded resources (e.g. R packages, Python packages, conda packages, etc.) (**Table 1**). Query and export of local and online databases (e.g. Pubmed and Datasets2tools) (Torre et al. 2018) with formatted tables are supported by BioInstaller that now not all web service provided the convenient interfaces to get the formatted table.

Massive bioinformatics tools or scripts are using the source code (e.g. .py, pl) to store the data analysis required information , such as URLs and versions of source code and databases (e.g. <https://sourceforge.net/projects/fusioncatcher/files/bootstrap.py>), which will reduce the reusability and compatibility for other programming platforms. Using the pre-defined files (e.g. TOML format meta databases and the plugins containing the R commands) and functions of BioInstaller, users can integrate these TOML format files storing the URLs of required tools or databases into their own pipelines.

**Plugin system of Shiny applications based on TOML files**

The functions customize of de novo Shiny applications are still relatively complicated that lack a plugin management and setting system similar to Galaxy (Afgan et al. 2016) or other cloud computing platforms such as Dnanexus (<https://www.dnanexus.com/>) and illumine BaseSpace (<https://basespace.illumina.com/>). Here, we implemented a lightweight plugins management and the setting module, which simultaneously includes the Shiny graphics interface and YAML editor to manage the plugins and global variables used by BioInstaller and its plugins. Several R packages (e.g. annovarR, Maftools, clusterProfiler, CEMiTool) (Mayakonda & Koeffler 2016; Russo et al. 2018; Yu et al. 2012), command line tools (e.g. VEP, vcfanno, ANNOVAR) (McLaren et al. 2016; Pedersen et al. 2016; Wang et al. 2010), online APIs (e.g. Pubmed, Datasets2Tools) (Torre et al. 2018), and custom functions (e.g. creating pre-defined bioinformatics data analysis directory tree) have been wrapped by BioInstaller plugins (<https://github.com/JhuangLab/BioInstaller/tree/master/inst/extdata/config/shiny> and https://github.com/JhuangLab/annovarR/tree/master/inst/extdata/config).

**REFERENCES**

Afgan E, Baker D, van den Beek M, Blankenberg D, Bouvier D, Cech M, Chilton J, Clements D, Coraor N, Eberhard C, Gruning B, Guerler A, Hillman-Jackson J, Von Kuster G, Rasche E, Soranzo N, Turaga N, Taylor J, Nekrutenko A, and Goecks J. 2016. The Galaxy platform for accessible, reproducible and collaborative biomedical analyses: 2016 update. *Nucleic Acids Res* 44:W3-W10. 10.1093/nar/gkw343

Mayakonda A, and Koeffler HP. 2016. Maftools: Efficient analysis, visualization and summarization of MAF files from large-scale cohort based cancer studies. *Available at* [*https://www.biorxiv.org/content/biorxiv/early/2016/05/11/052662.full.pdf*](https://www.biorxiv.org/content/biorxiv/early/2016/05/11/052662.full.pdf) (accessed Sep.3 2018).

McLaren W, Gil L, Hunt SE, Riat HS, Ritchie GR, Thormann A, Flicek P, and Cunningham F. 2016. The Ensembl Variant Effect Predictor. *Genome Biol* 17:122. 10.1186/s13059-016-0974-4

Pedersen BS, Layer RM, and Quinlan AR. 2016. Vcfanno: fast, flexible annotation of genetic variants. *Genome Biol* 17:118. 10.1186/s13059-016-0973-5

Russo PST, Ferreira GR, Cardozo LE, Bürger MC, Arias-Carrasco R, Maruyama SR, Hirata TDC, Lima DS, Passos FM, Fukutani KF, Lever M, Silva JS, Maracaja-Coutinho V, and Nakaya HI. 2018. CEMiTool: a Bioconductor package for performing comprehensive modular co-expression analyses. *BMC Bioinformatics* 19. 10.1186/s12859-018-2053-1

Torre D, Krawczuk P, Jagodnik KM, Lachmann A, Wang Z, Wang L, Kuleshov MV, and Ma'ayan A. 2018. Datasets2Tools, repository and search engine for bioinformatics datasets, tools and canned analyses. *Sci Data* 5:180023. 10.1038/sdata.2018.23

Wang K, Li M, and Hakonarson H. 2010. ANNOVAR: functional annotation of genetic variants from high-throughput sequencing data. *Nucleic Acids Res* 38:e164. 10.1093/nar/gkq603

Yu G, Wang LG, Han Y, and He QY. 2012. clusterProfiler: an R package for comparing biological themes among gene clusters. *OMICS* 16:284-287. 10.1089/omi.2011.0118
