# Supplementary material for: BioInstaller: a comprehensive R package to construct interactive and reproducible biological data analysis applications based on the R platform
Source: PeerJ. 2018 Oct 31;6:e5853. doi: 10.7717/peerj.5853 (PMC6215441; doi:10.7717/peerj.5853)
Supplement: Supplemental Information 7 — (A)(B) Using the download of ‘db_annovar_refgene’ shows how it works of Shiny ‘Installer’ module. Dynamic and interactive manipulations are supported. Log information of submitted download/install job can be recalled using given random characters. (C)(D) The input box and output log to create a new softwares environment using the conda plugin of ‘Installer’ module. (E)(F) The input box and output log to install ‘zlib’ using the spack plugin of ‘Installer’ module. [file peerj-06-5853-s007.pdf]

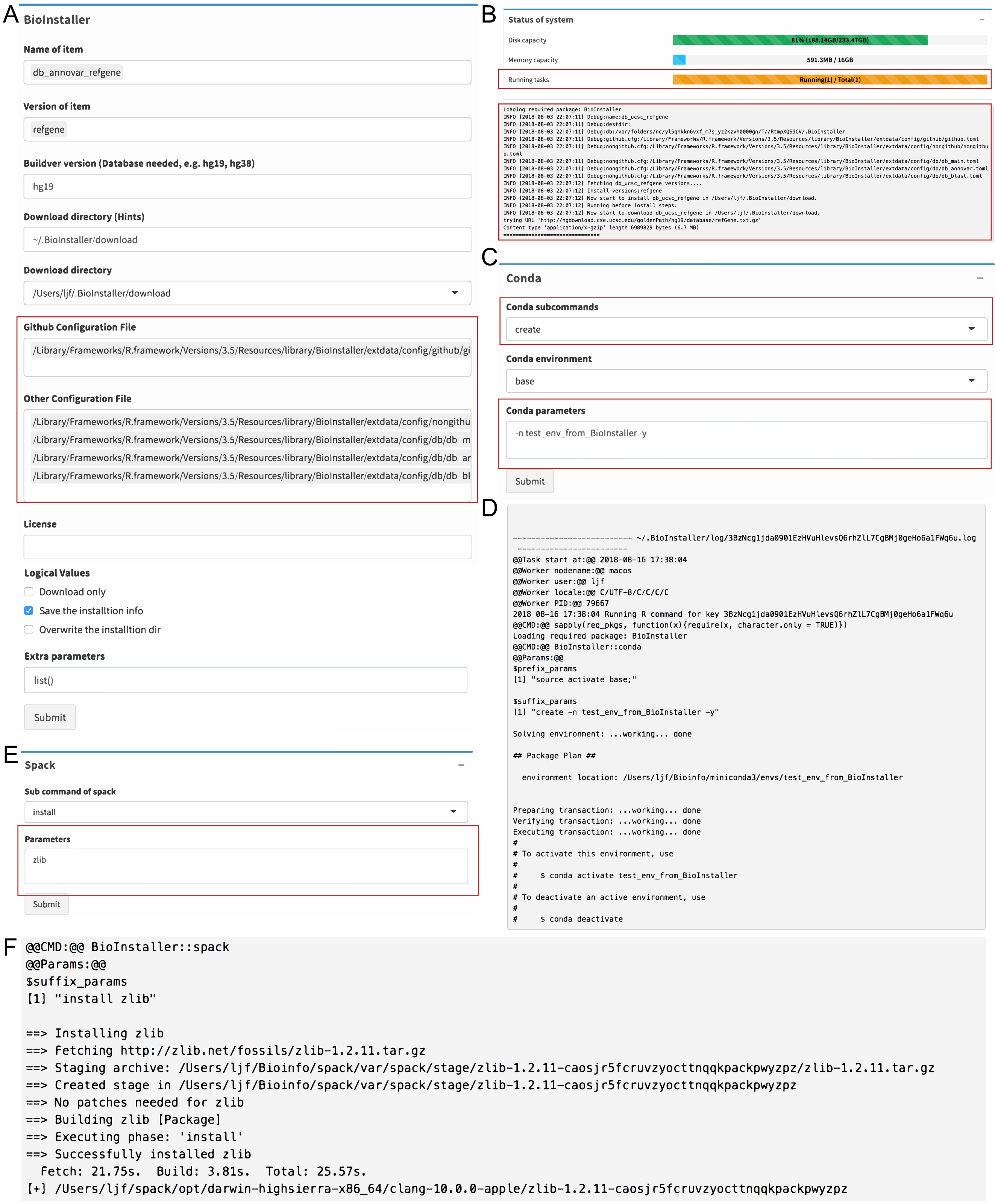

A
BioInstaller
Name of item
db_annovar_refgene
Version of item
refgene
Buildver version (Database needed, e.g. hg19, hg38)
hg19
Download directory (Hints)
~/.BioInstaller/download
Download directory
/Users/ljf/.BioInstaller/download
Github Configuration File
/Library/Frameworks/R.framework/Versions/3.5/Resources/library/BioInstaller/extdata/config/github/gi
Other Configuration File
/Library/Frameworks/R.framework/Versions/3.5/Resources/library/BioInstaller/extdata/config/nongithu
/Library/Frameworks/R.framework/Versions/3.5/Resources/library/BioInstaller/extdata/config/db/db_m
/Library/Frameworks/R.framework/Versions/3.5/Resources/library/BioInstaller/extdata/config/db/db_ar
/Library/Frameworks/R.framework/Versions/3.5/Resources/library/BioInstaller/extdata/config/db/db_bl
License
Logical Values
Download only
Save the installtion info
Overwrite the installtion dir
Extra parameters
list()
Submit
B
Status of system
Disk capacity
81% (188.14GB/233.47GB)
Memory capacity
591.3MB / 16GB
Running tasks
Running(1) / Total(1)
Loading required package: BioInstaller
INFO [2018-08-03 22:07:11] Debug:name:db_ucsc_refgene
INFO [2018-08-03 22:07:11] Debug:destdir:
INFO [2018-08-03 22:07:11] Debug:db:/var/folders/nc/yl5qhkkn6vxf_m7s_yz2kzvh0000gn/T//RtmpXQS9CV/.BioInstaller
INFO [2018-08-03 22:07:11] Debug:github.cfg:/Library/Frameworks/R.framework/Versions/3.5/Resources/library/BioInstaller/extdata/config/github/github.toml
INFO [2018-08-03 22:07:11] Debug:nongithub.cfg:/Library/Frameworks/R.framework/Versions/3.5/Resources/library/BioInstaller/extdata/config/nongithub/nongithu
b.toml
INFO [2018-08-03 22:07:11] Debug:nongithub.cfg:/Library/Frameworks/R.framework/Versions/3.5/Resources/library/BioInstaller/extdata/config/db/db_main.toml
INFO [2018-08-03 22:07:11] Debug:nongithub.cfg:/Library/Frameworks/R.framework/Versions/3.5/Resources/library/BioInstaller/extdata/config/db/db_annovar.toml
INFO [2018-08-03 22:07:11] Debug:nongithub.cfg:/Library/Frameworks/R.framework/Versions/3.5/Resources/library/BioInstaller/extdata/config/db/db_blast.toml
INFO [2018-08-03 22:07:12] Fetching db_ucsc_refgene versions....
INFO [2018-08-03 22:07:12] Install versions:refgene
INFO [2018-08-03 22:07:12] Now start to install db_ucsc_refgene in /Users/ljf/.BioInstaller/download.
INFO [2018-08-03 22:07:12] Running before install steps.
INFO [2018-08-03 22:07:12] Now start to download db_ucsc_refgene in /Users/ljf/.BioInstaller/download.
trying URL 'http://hgdownload.cse.ucsc.edu/goldenPath/hg19/database/refGene.txt.gz'
Content type 'application/x-gzip' length 6989829 bytes (6.7 MB)
==============================
C
Conda
Conda subcommands
create
Conda environment
base
Conda parameters
-n test_env_from_BioInstaller -y
Submit
D
------------------------- ~/.BioInstaller/log/3BzNcg1jda0901EzHVuHlevsQ6rhZlL7CgBMj0geHo6a1FWq6u.log
----
@@Task start at:@@ 2018-08-16 17:38:04
@@Worker nodename:@@ macos
@@Worker user:@@ ljf
@@Worker locale:@@ C/UTF-8/C/C/C/C
@@Worker PID:@@ 79667
2018 08-16 17:38:04 Running R command for key 3BzNcg1jda0901EzHVuHlevsQ6rhZlL7CgBMj0geHo6a1FWq6u
@@CMD:@@ sapply(req_pkgs, function(x){require(x, character.only = TRUE)})
Loading required package: BioInstaller
@@CMD:@@ BioInstaller::conda
@@Params:@@
$prefix_params
[1] "source activate base;"
$suffix_params
[1] "create -n test_env_from_BioInstaller -y"
Solving environment: ...working... done
Package Plan
environment location: /Users/ljf/Bioinfo/miniconda3/envs/test_env_from_BioInstaller
Preparing transaction: ...working... done
Verifying transaction: ...working... done
Executing transaction: ...working... done
#
To activate this environment, use
#
$ conda activate test_env_from_BioInstaller
#
To deactivate an active environment, use
#
$ conda deactivate
E
Spack
Sub command of spack
install
Parameters
zlib
Submit
F
@@CMD:@@ BioInstaller::spack
@@Params:@@
$suffix_params
[1] "install zlib"
==> Installing zlib
==> Fetching http://zlib.net/fossils/zlib-1.2.11.tar.gz
==> Staging archive: /Users/ljf/Bioinfo/spack/var/spack/stage/zlib-1.2.11-caosjr5fcruvzyocttnqqkpackpwyzpz/zlib-1.2.11.tar.gz
==> Created stage in /Users/ljf/Bioinfo/spack/var/spack/stage/zlib-1.2.11-caosjr5fcruvzyocttnqqkpackpwyzpz
==> No patches needed for zlib
==> Building zlib [Package]
==> Executing phase: 'install'
==> Successfully installed zlib
Fetch: 21.75s. Build: 3.81s. Total: 25.57s.
[+] /Users/ljf/spack/opt/darwin-highsierra-x86_64/clang-10.0.0-apple/zlib-1.2.11-caosjr5fcruvzyocttnqqkpackpwyzpz
