# Supplementary material for: BioInstaller: a comprehensive R package to construct interactive and reproducible biological data analysis applications based on the R platform
Source: PeerJ. 2018 Oct 31;6:e5853. doi: 10.7717/peerj.5853 (PMC6215441; doi:10.7717/peerj.5853)
Supplement: Supplemental Information 9 — (A) Search the keyword on the github website with the the autosuggestor autocompletion functionality (B) Demo output of github search using ‘RNA’. Key word was colored yellow. (C) Demo posts of forum of BioInstaller. [file peerj-06-5853-s009.pdf]

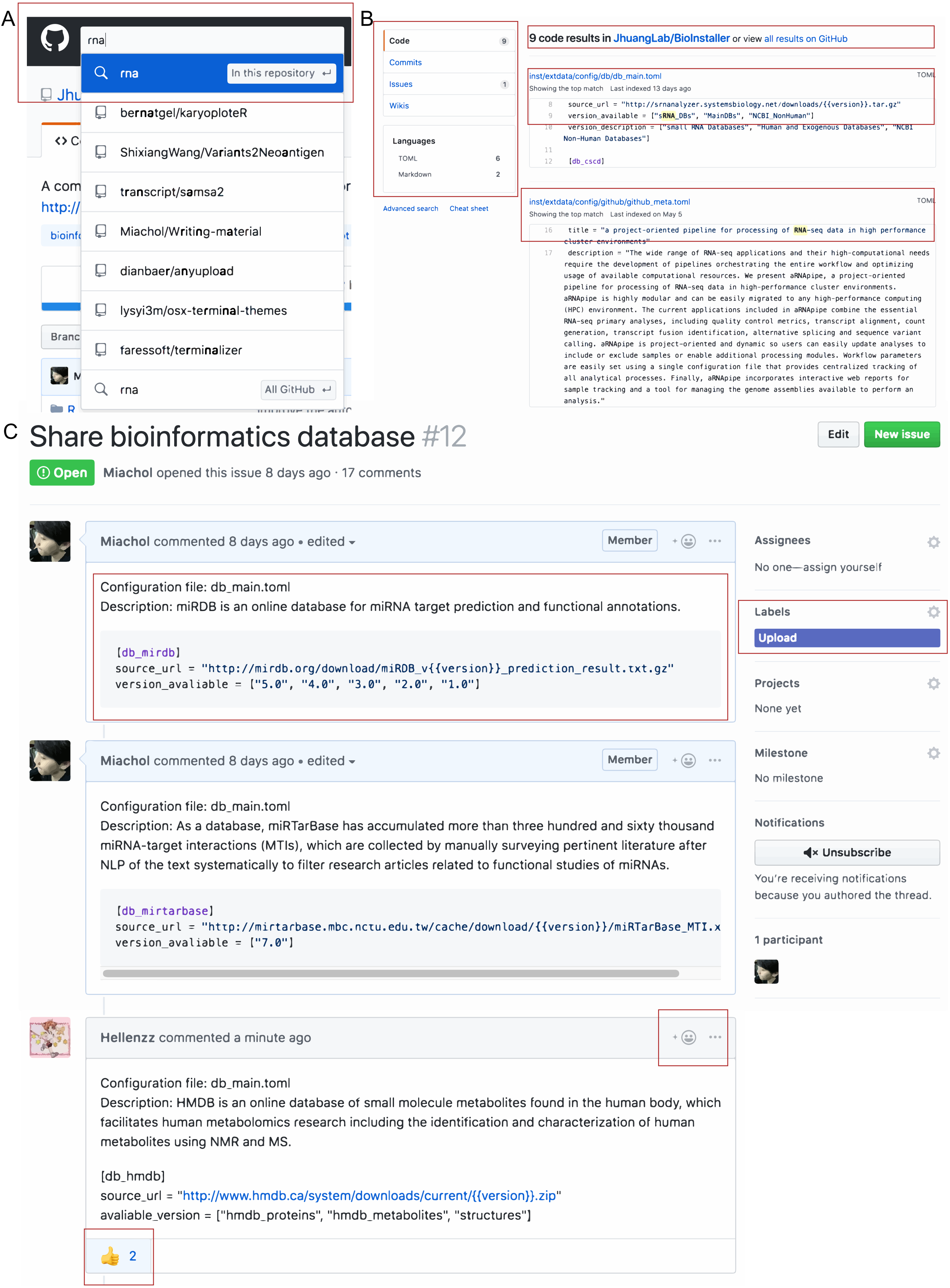
A
rna
rna In this repository
bernatgel/karyoploteR
ShixiangWang/Variants2Neoantigen
transcript/samsa2
Miachol/Writing-material
dianbaer/anyupload
lysyi3m/osx-terminal-themes
faressoft/terminalizer
rna All GitHub
B
Code 9
Commits
Issues 1
Wikis
Languages
TOML 6
Markdown 2
Advanced search
Cheat sheet
9 code results in JhuangLab/BioInstaller or view all results on GitHub
inst/extdata/config/db/db_main.toml
TOML
Showing the top match Last indexed 13 days ago
8 source_url = "http://srnanalyzer.systemsbiology.net/downloads/{{version}}.tar.gz"
9 version_available = ["sRNA_DBs", "MainDBs", "NCBI_NonHuman"]
10 version_description = ["small RNA Databases", "Human and Exogenous Databases", "NCBI Non-Human Databases"]
11
12 [db_cscd]
inst/extdata/config/github/github_meta.toml
TOML
Showing the top match Last indexed on May 5
16 title = "a project-oriented pipeline for processing of RNA-seq data in high performance cluster environments"
17 description = "The wide range of RNA-seq applications and their high-computational needs require the development of pipelines orchestrating the entire workflow and optimizing usage of available computational resources. We present aRNApipe, a project-oriented pipeline for processing of RNA-seq data in high-performance cluster environments. aRNApipe is highly modular and can be easily migrated to any high-performance computing (HPC) environment. The current applications included in aRNApipe combine the essential RNA-seq primary analyses, including quality control metrics, transcript alignment, count generation, transcript fusion identification, alternative splicing and sequence variant calling. aRNApipe is project-oriented and dynamic so users can easily update analyses to include or exclude samples or enable additional processing modules. Workflow parameters are easily set using a single configuration file that provides centralized tracking of all analytical processes. Finally, aRNApipe incorporates interactive web reports for sample tracking and a tool for managing the genome assemblies available to perform an analysis."
C
Share bioinformatics database #12
Edit
New issue
Open
Miachol opened this issue 8 days ago · 17 comments
Miachol commented 8 days ago • edited
Member
Configuration file: db_main.toml
Description: miRDB is an online database for miRNA target prediction and functional annotations.
[db_mirdb]
source_url = "http://mirdb.org/download/miRDB_v{{version}}_prediction_result.txt.gz"
version_avaliable = ["5.0", "4.0", "3.0", "2.0", "1.0"]
Miachol commented 8 days ago • edited
Member
Configuration file: db_main.toml
Description: As a database, miRTarBase has accumulated more than three hundred and sixty thousand miRNA-target interactions (MTIs), which are collected by manually surveying pertinent literature after NLP of the text systematically to filter research articles related to functional studies of miRNAs.
[db_mirtarbase]
source_url = "http://mirtarbase.mbc.nctu.edu.tw/cache/download/{{version}}/miRTarBase_MTI.x
version_avaliable = ["7.0"]
Hellenzz commented a minute ago
Configuration file: db_main.toml
Description: HMDB is an online database of small molecule metabolites found in the human body, which facilitates human metabolomics research including the identification and characterization of human metabolites using NMR and MS.
[db_hmdb]
source_url = "http://www.hmdb.ca/system/downloads/current/{{version}}.zip"
avaliable_version = ["hmdb_proteins", "hmdb_metabolites", "structures"]
2
Assignees
No one—assign yourself
Labels
Upload
Projects
None yet
Milestone
No milestone
Notifications
Unsubscribe
You're receiving notifications because you authored the thread.
1 participant
